# The Endangered Sardinian Grass Snake: Distribution Update, Bioclimatic Niche Modelling, Dorsal Pattern Characterisation, and Literature Review

**DOI:** 10.3390/life13091867

**Published:** 2023-09-04

**Authors:** Matteo Riccardo Di Nicola, Andrea Vittorio Pozzi, Sergio Mezzadri, Francesco Paolo Faraone, Giorgio Russo, Jean Lou M. C. Dorne, Gianmarco Minuti

**Affiliations:** 1 Faculty of Veterinary Medicine, Department of Pathobiology, Pharmacology and Zoological Medicine, Wildlife Health Ghent, Ghent University, 9820 Merelbeke, Belgium; 2 Unit of Dermatology and Cosmetology, IRCCS San Raffaele Hospital, Via Olgettina 60, 20132 Milan, Italy; 3 Asociación Herpetológica Española, Apartado de Correos 191, 28911 Leganés, Spain; 4 Molecular Ecology and Evolution Group, School of Natural Sciences, Bangor University, Bangor LL57 2UW, UK; pozzi.andrea95@outlook.com; 5 Independent Researcher, Via Palmerio, 29121 Piacenza, Italy; sergio.mezzadri@libero.it; 6 Dipartimento Scienze e Tecnologie Biologiche, Chimiche e Farmaceutiche, University of Palermo, Via Archirafi 18, 90123 Palermo, Italy; francescopaolo.faraone@unipa.it; 7 VIB-VUB Center for Structural Biology, Vrije Universiteit Brussel, 1090 Brussels, Belgium; giorgio.russo92@gmail.com; 8 Methodology and Scientific Support Unit, European Food Safety Authority (EFSA), Via Carlo Magno 1A, 43126 Parma, Italy; jean-lou.dorne@efsa.europa.eu; 9 Ecology & Biodiversity Research Unit, Department of Biology, Vrije Universiteit Brussels, 1090 Brussels, Belgium; gianmarco.minuti@outlook.it

**Keywords:** Colubridae, habitat suitability, island endemic, Natricidae, *Natrix*, *helvetica*, *cetti*, potential distribution, Reptilia, Sardinia

## Abstract

The Sardinian grass snake, *Natrix helvetica cetti*, is an endangered endemic snake subspecies with a restricted and highly fragmented geographic distribution. Information on its ecology and detailed geographic distribution are scarce and may negatively impact on its conservation status. Therefore, a literature review on its taxonomy, morphology, ecology, and conservation is presented here. Moreover, field records from the authors, citizen science and the existing literature provide an updated geographic distribution highlighting its presence within 13 new and 7 historic 10 × 10 km cells. Bioclimatic niche modelling was then applied to explore patterns of habitat suitability and phenotypic variation within *N. h. cetti*. The geographic distribution of the species was found to be positively correlated with altitude and precipitation values, whereas temperature showed a negative correlation. Taken together, these outcomes may explain the snake’s presence, particularly in eastern Sardinia. In addition, analysis of distribution overlap with the competing viperine snake (*N. maura*) and the urodeles as possible overlooked trophic resources (*Speleomantes* spp. and *Euproctus platycephalus*) showed overlaps of 66% and 79%, respectively. Finally, geographical or bioclimatic correlations did not explain phenotypic variation patterns observed in this highly polymorphic taxon. Perspectives on future research to investigate *N. h. cetti*’s decline and support effective conservation measures are discussed.

## 1. Introduction

Endemism, together with species rarity and diversity, are among the most used criteria to establish conservation priorities [1,2]. Habitat alteration, pollution, climate change, and introduction of invasive species are the main threats to endemics, which are highly adapted to their specific habitats [2,3,4,5].

Among the factors affecting the probability of extinction of a species, rarity is the predominant one, and species become rare before going extinct. Hence, endemic species with a limited distribution range and low dispersal rate are most susceptible to extinction [2]. It is, therefore, important to focus efforts on the conservation of endemic taxa, and one of the preliminary steps is to assess their known and potential distribution in order to be able to detect populations and consequently study them appropriately. Indeed, understanding species distribution is fundamental to tracking and predicting changes in range dynamics and thus setting conservation priorities [6]. Nevertheless, this process is complex and in order to provide meaningful and practical conservation insights, it requires taking into consideration multiple layers of information in addition to pure presence/absence data [7,8,9]. This allows us to better understand the real extent of the geographic range of cryptic and rare species, overcoming the constraints correlated with field surveys, and in some cases, leading to the discovery of new and valuable populations (e.g., [10]).

**Figure 1:**
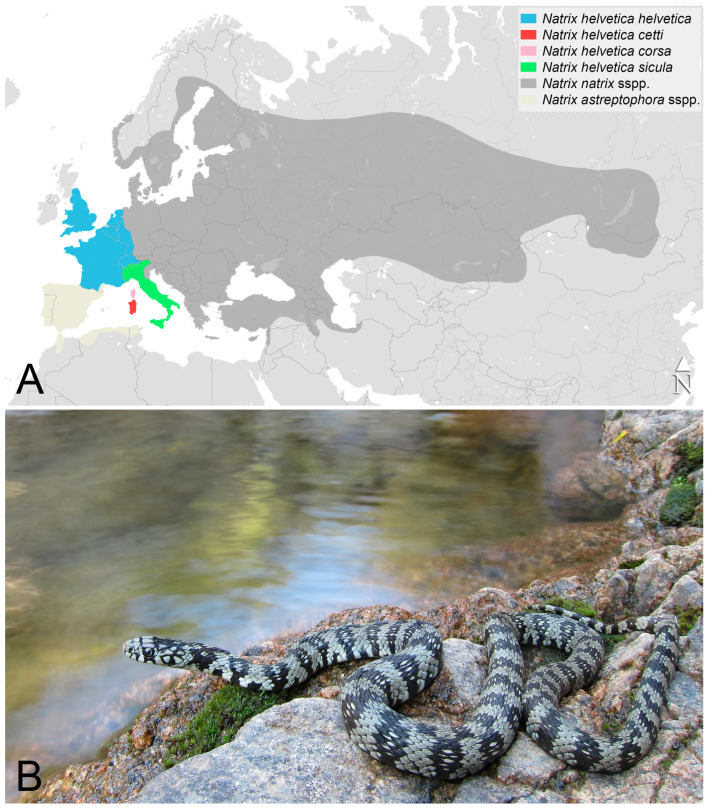
Western and Central Eurasia, with the approximate* distribution ranges of the *Natrix natrix* complex species and *N. helvetica* subspecies according to [21,22,23,29]. *Admixture areas between different taxa are not shown (**A**); Adult individual of *Natrix helvetica cetti* in its natural habitat in the “Sette Fratelli” area, South Sardinia (**B**). Photo credit: Matteo R. Di Nicola.

The island of Sardinia (in red in Figure 1A) is situated in the central-western part of the Mediterranean Sea, and it is the second largest island of the Mediterranean Basin (extension: 23,812.6 km^2^; max altitude: 1834 m a.s.l. [11]) where, together with Corsica, it is one of the major biodiversity hotspots [12,13]. As for the herpetofauna, Sardinia has 19 species of reptiles, including the loggerhead sea turtle *Caretta caretta* since it is nesting on the island (see Table 1; [12,14,15,16]). The Sardinian snake fauna consists of four species, half of which share a very recent origin from North Africa, determined by probable introductions in historical times. These are the horseshoe whip snake *Hemorrhois hippocrepis* [17] and the viperine snake *Natrix maura* (for the latter, even a transmarine dispersal cannot be excluded; see [18] and Section 3.1.4). The other two snake species have colonised the Sardinian-Corsican block from the Italian peninsula in relatively recent times. These are the western whip snake *Hierophis viridiflavus* (see [19]) and the barred grass snake *N. helvetica*. The Sardinian-Corsican populations of *N. helvetica* then differentiated from the continental ones, resulting in a separate subspecific taxon [20,21]. 

The Sardinian grass snakes and the Corsican grass snakes could be considered synonyms since they have been demonstrated to be genetically undifferentiated [22]. However, pending studies include a larger sample from both islands, and considering the high threat status of the Sardinian populations, it has been suggested to tentatively keep two separate subspecies -*N. h. cetti* and *N. h. corsa*-([22,23]; see Section 3.1.1). For this reason, in the present work, the taxon name *N. h. cetti* is applied in reference to the Sardinian populations only. 

*Natrix h. cetti* (Figure 1B) is irregularly distributed only on Sardinia’s main island and is considered to be in decline, with a smaller distribution than in the past [22,23,24,25], as well as being categorised as “Endangered” in the last IUCN Red List of Italian vertebrates [26]. 

Low detectability of individuals (possibly due to their elusiveness, limited distribution, and rarefaction) and the lack of proper field surveys are factors that could have negatively influenced the knowledge of the distribution of *N. h. cetti* [24,25]. The major aim of this work is to provide updated distribution data as well as an estimate of the potential distribution for the Sardinian grass snake. These aspects are fundamental to assess the current status of the populations and to potentially better understand the ecology of the taxon in order to refine conservation efforts.

Firstly, the available information on the various zoological aspects of the taxon is collected from a range of literature sources and presented here. This work may thus be used as a starting point for conservation planning. Furthermore, updated distribution data derived from personal field surveys and citizen science as well as potential distribution data obtained using bioclimatic niche modelling are presented. In addition, the range overlap of *N. h. cetti* and that of *N. maura*, a potential ecological competitor that colonized the island relatively recently (see [18,27] and Section 3.1.4), is also considered as a starting point to evaluate how much, from a spatial point of view, the presence of the non-native taxon may pose a threat to the insular endemic grass snake. Similarly, the range overlap of *N. h. cetti* with that of the six Sardinian endemic urodelan species (*Speleomantes* spp. and *Euproctus platycephalus*) is assessed, as the latter could represent an overlooked trophic resource for the Sardinian grass snake (see Section 3.1.3). Finally, an attempt is made to characterise the variability in the dorsal pattern (i.e., the dark marks on the ground colour), given that the only work concerning chromatic variability of the Sardinian grass snake essentially referred to the dorsal ground colouration (see [28]). The influence of distributional and environmental variables on dorsal pattern variation is also investigated.

## 2. Materials and Methods

### 2.1. Natural History Review

All available literature on the Sardinian grass snake (i.e., scientific articles, thematic books, and guides) was gathered by consulting the PubMed and Scopus databases, the social network ResearchGate, and by using the Google Scholar web search engine.

To perform the search, the following query was used, applying every possible combination of the following keywords: [<Sardinia> OR <Sardinian> AND <snakes> OR <reptiles> OR <herpetofauna> OR (<*grass*> AND <*snake*>)] OR [<*Natrix*> OR <*Tropidonotus*> AND <*cetti*> OR <*cettii*> OR (<*natrix*> AND <*cetti*>) OR (<*natrix*> AND <*cettii*>) OR (<*natrix*> AND <*corsus*>) OR (<*natrix*> AND <*corsa*>) OR (<*helvetica*> AND <*cetti*>) OR (<*helvetica*> AND <*corsa*>)].

The most relevant information found in the literature was summarised to compose a brief review divided into taxonomy and hypothesis on the origins, morphology, ecology, and conservation.

### 2.2. Distribution Update

The distribution update of *Natrix helvetica cetti* in Sardinia was produced on the basis of field observations carried out by two of the authors (MRDN and SM) during opportunistic surveys conducted in the period July 2016–May 2022 (33.3%), and combined with data obtained from citizen science, updated until May 2023 (66.7%). For each snake found by the authors, the date, time, coordinates, and altitude were recorded (via Garmin Etrex 32X GPS device), and a photo of the dorsal pattern was taken. As for the records obtained through citizen science, the data were requested from herpetologist/herpetophile collaborators and observations were also sought on the following Social Networks: Inaturalist.it, Flickr.com, and the Facebook group “Identificazione Anfibi e Rettili”, administered by MRDN. The authors of each observation were then contacted, and only the records with coordinates recorded live (or for which they remembered the place of discovery with an accuracy of at least 300 m) and accompanied by a photo with sufficient resolution were considered. The altitudes of the citizen science records were obtained using the Google Earth Software (ver. 9.194.0.0).

The obtained records were then compared and added to those present in the scientific literature (i.e., [12,14,30,31,32,33,34,35]).

The new data were used to compile both a point map and an updated distribution map-UTM (Universal Transverse Mercator Projection, Coordinate Reference System WGS84/UTM Zone 32N) 10 × 10 km square grid system, which divided the island into 312 squares, similar to the one used by Corti et al. [12].

The *N. h. cetti* updated UTM distribution map was then overlaid with the map for *N. maura*. The latter was obtained by merging the data published by Corti et al. [12] with the records collected by two of the authors (MRDN and SM) and citizen science.

Furthermore, the *N. h. cetti* updated UTM distribution map was also overlaid on those from the six Sardinian species of urodeles (*Speleomantes* spp. and *Euproctus platycephalus*), based on data from the publication by Corti et al. [12].

### 2.3. Habitat Suitability Estimation

The software MaxEnt 3.4.1 [36] was used to model the bioclimatic niche of *Natrix helvetica cetti* in Sardinia. Only occurrence records with precise coordinates were used for this purpose. In order to avoid pseudo-replication, these were spatially filtered to retain one record per grid cell (30 arcsec spatial resolution). Predictor layers were composed of 19 bioclimatic variables plus altitude downloaded from the WorldClim 2.1 database [37], and a land cover raster obtained from the Global Land Cover National Mapping Organizations (GLCNMO version 3 available at https://globalmaps.github.io/glcnmo.html, accessed on 5 June 2023). The bioclimatic variables were downloaded at a 30 arc-sec spatial resolution and rescaled to match the resolution of the land cover layer (15 arc-sec). Values for all 19 bioclimatic variables were extracted at each presence location and pairwise Pearson’s correlation coefficients were calculated in order to assess multicollinearity between predictors. Among highly correlated variables (|*r*| > 0.75), those deemed as most ecologically meaningful for the distribution of the taxon were retained, while the others were dropped. The selected variables were: mean annual temperature (bio1), temperature seasonality (bio4), annual precipitation (bio12), precipitation seasonality (bio15), and precipitation of the warmest quarter (bio18). Altitude and land cover were added in a step-wise procedure to observe their effect on model output. In order to assess the influence of variable selection on the models, we compared the performances of our dataset before and after variable selection. A total of six model combinations were tested: (1) all bioclimatic variables (n = 19); (2) bioclimatic variables plus altitude (n = 20); (3) bioclimatic variables plus altitude and land cover (n = 21); (4) only selected bioclimatic variables (n = 5); (5) selected bioclimatic variables plus altitude (n = 6); (6) selected bioclimatic variables plus altitude and land cover (n = 7). A mask layer of Sardinia was used to draw background points (n = 10,000) for modelling in MaxEnt (default settings; logistic outputs). Ten bootstrap replicates were computed for every model combination, each replicate randomly selecting 70% of the data points for model training and the remaining 30% for validation. Jackknife analysis was used to estimate the relative contribution of each predictor variable to the models. A model’s performance was evaluated based on the area under the receiver operating curve (AUC) and omission rate (OR). The former is a measure of the model’s ability to discriminate between presence and background points and can range from 0.5 (no better than chance) to 1 (perfect discrimination; [38]). The latter expresses the proportion of records predicted to fall outside the area predicted as suitable by the model, based on various theoretical thresholds. For the purpose of this study, the 10th percentile training presence omission rate (OR10) was adopted. This sets the minimum suitability threshold at a value allowing only 10% of possible predicted occurrences to be rejected; models that are significantly higher that OR10 are, therefore, considered overfit [39]. The OR10 logistic threshold was also used to set the minimum suitability threshold to MaxEnt continuous suitability outputs. Finally, an average of the six model combinations tested was computed and used to represent the bioclimatic suitability of *N. h. cetti* in Sardinia. All analyses were performed in R 3.6.1 [40] and QGIS 3.14 [41].

### 2.4. Dorsal Pattern Characterisation

The Sardinian grass snake is characterised by a high level of colour polymorphism (e.g., [15,25,28]). In reptiles, this interspecific colour variation may be correlated with geographic, bioclimatic, and environmental factors (e.g., [42,43,44]). Therefore, we carried out a tentative characterisation of the dorsal patterns, based on both personally acquired and citizen science photographs (the unavailability of photos taken in a standardized way for all snakes, has not allowed the use of computer-based colour assessment methods). In this endeavour, only observations with photographs deemed suitable for the purpose were considered (i.e., with sufficient resolution, correctly exposed, and showing most of the dorsal pattern of the snake). Since the colouration in *Natrix natrix* sensu lato is possibly subject to ontogenetic variations (see [45]; e.g., melanism in *N. helvetica* becomes apparent in adulthood-Faraone, unpublished data), we excluded individuals identified as clearly young (i.e., newborn or no more than yearling, based on body proportions, such as the size of the eyes in relation to the head and the size of the latter in relation to the body) from the evaluation. In some cases, the dorsal pattern does not unequivocally fall into a given category. Hence, we considered the prevailing trend of each dorsal motif and the evaluation was made independently by three of the authors (MRDN, AP, and SM), and this was followed by a collective assessment to discuss any discordant data. Overall, the dorsal patterns of snakes that had coordinates were geographically mapped and the distribution of the different dorsal patterns was visualised using the 10 × 10 km UTM map described in Section 2.2.

Dorsal pattern characterisation was used to explore patterns of geographic segregation and to assess the influence of bioclimatic variables via principal component analysis (see the next section).

### 2.5. Principal Component Analysis

A principal component analysis (PCA) was computed in order to explore the bioclimatic niche occupied by individuals of *Natrix helvetica cetti* in Sardinia, differentiating them across various dorsal patterns. To do so, both presence and background points used to compute the above-mentioned MaxEnt models (see Section 2.3) were plotted across the first two principal component dimensions, representing their variation along the reduced set of continuous variables used for modelling (bio1, bio4, bio12, bio15, and bio18). The statistical analyses and visual representation were performed in R 3.6.1 using the packages *FactoMineR* [46], *factoextra* [47] and *ggplot2* [48].

## 3. Results

### 3.1. Natural History Review

#### 3.1.1. Taxonomy and Hypothesis on the Origins

Grass snakes (*Natrix natrix* complex) are medium-sized to large semi-aquatic Natricidae ophidians distributed from the Maghreb region and the Iberian Peninsula throughout most of Europe to Lake Baikal in central Asia ([21,23,49,50]; Figure 1A). Three different parapatric species are currently recognised [the red-eyed or Iberian grass snake *Natrix astreptophora* (Seoane, 1884); the barred grass snake *Natrix helvetica* (Lacépède, 1789); and the common or Eastern grass snake *Natrix natrix* (Linnaeus, 1758)], and between their distribution ranges, there are narrow hybridization areas in which both hybrids and parental species are present [22,51,52,53,54,55,56,57]. Different subspecies have been described within the *N. natrix* complex, and Fritz and Schmidtler [23] and Fritz and Ihlow [57] have provided a list of those currently considered valid (Table S1).

The grass snakes from Sardinia and Corsica have been described as a new species *Natrix cetti* by Gené [58] on the basis of an adult male from Monte di San Giovanni d’Iglesias (southern Sardinia), a pregnant female from Fonni (central Sardinia), and a juvenile from southern Sardinia Corsica (see [23] for more information on syntypes). Mertens and Muller [59] restricted the type locality of the species only to the Monte di San Giovanni d’Iglesias. Hence, *N. cetti* has generally been considered “the grass snake from Sardinia”. Anyway, the authors did not designate a lectotype and their restriction of the type locality is not valid: the lectotype will then be designated only by Fritz and Schmidtler [23], starting from the female syntype from Fonni. Leunis [60] ascribed the species to the genus *Tropidonotus* Boie, 1826, while Jan [61], Camerano [62], and Mertens and Müller [59] considered it a subspecies, while reusing the genus *Natrix*. Hecht [63], in a review of the genus *Tropidonotus*, considered the Corsican populations as a separate subspecies *Tropidonotus natrix* subsp. *Corsus*. Mertens [64] puts the Corsican taxon back into synonymy with the Sardinian one but later [65], investigating further samples from Corsica, reconsidered the separation of the subspecies (using *Natrix natrix corsa*). Roger S. Thorpe, starting from a PhD project on the intraspecific variation in *N. natrix* [66], carried out a review of the species based on biometrics. The author recognised a total of three subspecies for *N. natrix* (i.e., *N. n. natrix*, *N. n. helvetica* and *N. n. cetti*), considering the Corsican populations “phenetically intermediate” between the Sardinian and the continental ones. Hence, in this analysis, the two Sardinian-Corsican taxa were kept partially separate, indicating *N. n. cetti* for Sardinia and “*N. n. helvetica*-*N. n. cetti*” for Corsica ([67]; see also [68]). Later, the same author produced a more comprehensive work in which he recognised four *N. natrix* subspecies (i.e., *N. n. natrix*, *N. n. helvetica*, *N. n. cetti* and *N. n. corsa*) and reconfirmed the separation between the Sardinian and Corsican populations [45]. Still Thorpe [69], stated the following: “The four main forms, eastern, western Sardinian and Corsican, although treated as conspecific are obviously not far below the level of species”. In Lanza [70], a work by Vanni and Lanza indicated as “in preparation” (but no longer published in any other form) proposed the taxon *N. n. cetti* for both Sardinia and Corsica, following the examination of more than 800 Italian specimens as well as material from other regions. Based on the existing morphological and biogeographic differences from the other *N. natrix* and following the karyological data presented by Aprea et al. [71], Vanni and Cimmaruta [24] proposed the specific status for Sardinian-Corsican populations. Furthermore, given the morphological and chromatic differences between the Sardinian and Corsican populations, the authors indicated a possible subspecific distinction (with the names *N. cetti cetti* and *N. c. corsa*); although, this conclusion would still require confirmation at the molecular level. However, studies on a molecular basis have not confirmed the hypothesis of specific status: Fritz et al. [20] analysed Sardinian and Corsican mitochondrial DNA sequences, comparing them with sequences from the rest of the range, to reach the conclusion that the subspecific level has to be reconsidered for Sardinian-Corsican populations. Moreover, phylogenetic analyses by Kindler et al. [72] show that the two island populations share the same mtDNA lineage (named “B”), and they both fall into the third of the three major clades identified [i.e., (i) Iberian Peninsula, adjacent France and North Africa; (ii) East Europe and Asia; (iii) West Europe including Corso-Sardinia, the Apennine Peninsula and Sicily]. A study by Kindler et al. [52] raised *N. natrix helvetica* to the species level: since the recognition of *N. helvetica* as a full species implies that all nominal subspecies assigned to the same major clade (the third major clade of [72]) have to be transferred to *N. helvetica*, the Sardinian and Corsican grass snakes have been involved in the consequent taxonomic update, passing respectively from *N. natrix cetti* and *N. n. corsa* to *N. helvetica cetti* and *N. h. corsa*.

Further molecular analyses show that *N. h. cetti* and *N. h. corsa* constitute together only one microsatellite cluster [22]. Hence, the two taxa are considered genetically undifferentiated with respect to both nuclear and mitochondrial markers and could be synonymous (and they should, therefore, be considered a single Sardinian-Corsican endemic taxon). Overall, from the studies presented above, few samples from Sardinian and Corsican snakes were analysed and since the Sardinian grass snake is the most seriously threatened and apparently less abundant than its Corsican counterpart, a synonymisation of the two taxa could have a negative impact on the conservation of the Sardinian populations. For these reasons, the authors tentatively recognised both subspecies, referring to the need for further research [22,23].

Natricids likely originated ~35–47.1 million years ago in Asia [50,73,74], where most of the extant Natricidae species live, and dispersed to Australo-Melanesia, sub-Saharan Africa, Europe/North Africa, and North/Central America [74]. The *Natrix* lineage dispersed to the western Palearctic, where the genus *Natrix* diversified [21]. According to Deepak et al. [74], extant western Palearctic natricids started to diverge ~ 26 Mya, and extant *Natrix* spp. ~16 Mya with *N. maura*. Schöneberg et al. [21], who first used a genomics approach for the genus *Natrix*, indicate for the extant *Natrix* spp. the following divergence ages: *N. maura* ~21.5 Mya; *N. tessellata* ~18.7 Mya; *N. natrix* ~13.7 Mya; *N. astreptophora* split from *N. helvetica* ~7.3 Mya.

With regards to the origins of the Sardinian-Corsican taxon, Lanza [75,76,77] hypothesised a Messinian provenance (~5,2 Mya), or even a pre-Miocene one, whether the taxon is considered as full species; instead, arrival in the Quaternary is hypothesised during the Cassia regression (~1 Mya) if the taxon is considered at a subspecific level [24]. Fritz et al. [20], based on phylogenetic analysis, indicate a divergence from the continental forms between 4.4 and 4.3 Mya, during the lower Pliocene, probably derived from the isolation due to the post-Messinian floods of the Mediterranean basin, which occurred 5.33 Mya (see [78]). The most recent genomic-based phylogenetic analysis confirmed that the divergence would have occurred in the Pliocene, ~4.5 Mya [21]. On the other hand, the Sardinian and Corsican populations appear to be weakly differentiated from each other [20,22]. Fritz et al. [20] indicated an estimated mean time of divergence between 0.41 and 0.35 mya, finding a possible explanation in the intermittent land connections between the two islands, caused by sea level fluctuations in the Pleistocene (according to [79]). Similarly, Schultze et al. [22] argued that Sardinia and Corsica were repeatedly connected during the Pliocene and during the Pleistocene low sea-level stands (see [80]) and that the last connection between the two islands was interrupted by the rising sea in the early Holocene. Overall, from the body of evidence presented above, Corsican and Sardinian grass snakes have most likely repeatedly formed a contiguous population system and their morphological differences may derive from a recent divergence.

#### 3.1.2. Morphology

As highlighted by various authors (e.g., [24,28,45,69]), *Natrix helvetica cetti* includes grass snake populations with highly distinctive phenotypes. The chromatic pattern presents a peculiar expansion and fusion of its dark elements. Unlike other populations of *N. helvetica*, the dorsal and lateral series of transverse blotches often tend to merge and form large rings that split on the sides [28,45]. The neck is usually adorned with a large dark ring and the typical yellow or whitish nuchal collar, often present in mainland populations, is completely missing, even in the youngest individuals [24,45]. A large and irregular dark spot is generally present in the area of the parietal scales; furthermore, the remaining cephalic scales have dark edges of variable thickness. Cases of melanism are known for the Sardinian grass snake [15,25,28,81,82].

The Sardinian grass snake also possesses some highly distinctive pholidotic characters [45,69]. Ventral scales have a range of 160–178 in males and 158–173 in females [24] and are on average fewer than those from mainland populations [45]. Subcaudal scales are 56–65 in males and 47–53 in females ([24]; Di Nicola, unpublished records). This is much fewer than those found in mainland populations, which on average have about ten more units in both sexes [24,45]. On average, in Sardinian grass snakes, the sublabials and temporal scales are also about one unit less than those from mainland grass snakes [24,45].

Body proportions in *N. h. cetti* are different compared to those from other adjacent populations of barred grass snakes, which are characterised by proportionally smaller head characters, a thinner body, and a much shorter tail [45]. With regards to body size, Sardinian grass snakes usually do not reach 100 cm in total length [24,62,83], with a maximum of 109.5 cm for a female, as reported by Di Nicola and Mezzadri [15]. The total length of this subspecies is, therefore, smaller than that of mainland populations, reaching about 100 cm in males and 180 cm in females [25,84].

*Natrix helvetica cetti* also possesses some unique features of the internal organs, which are generally placed in a more caudal position compared to that of Italian mainland samples [45]. Amongst other differences, the right lung is considerably longer in the Sardinian population and the hemipenial retractor muscle much shorter compared to that reported for the other samples. Finally, the dentition of *N. h. cetti* have generally lower values than Italian mainland population [45].

#### 3.1.3. Ecology

The vast majority of the ecological aspects of the Sardinian grass snake are extremely understudied. The reasons could be the low density at which this snake seems to occur in the wild, its secretive nature, or most likely, a combination of both factors [25,83,85].

According to some authors, the ecology of *Natrix helvetica cetti* differs substantially from its mainland counterparts. Sardinian endemism seems to be less associated with water bodies, while it seems to inhabit rocky and dry habitats characterised by scarce vegetation and relevant sun exposure [86,87]. In agreement with the saxicolous nature of *N. h. cetti*, Lunghi and colleagues [28] observed 15 specimens (out of a total sample size of 18 individuals) within rocky habitats at a distance greater than 1 km from any water sources. On the other hand, Capula et al. [83], in a 38-day field study with a total sample size of 18 specimens, didn’t find any snakes further than 10 m away from water bodies such as mountain streams and lakes. It is then plausible that *N. h. cetti* is able to exploit a wider range of habitats compared to its mainland relatives [25,28]. The Sardinian grass snake is also considered to be primarily restricted to mid-elevation montane areas [34,86]. Despite this fact, individuals have been found from the sea level up to 1407 m a.s.l. ([15,28]; *this study*).

Another interesting aspect of the ecology of *N. h. cetti,* which might contribute to the knowledge gap that characterises this snake, regards its activity pattern. While grass snakes (*N. natrix* s. l.*)* tend to be predominantly diurnal, shifting to a nocturnal activity pattern during the hottest part of the year, *N. h. cetti* seems to be characterised by a nocturnal lifestyle [83]. In fact, despite searching for *N. h. cetti* in early spring and late summer (April and September), Capula and colleagues [83] only observed four individuals during the daytime, each of which was found hiding under natural refugia. The authors highlighted how active individuals were found at night-time while looking for potential prey during early spring at air temperatures lower than 15 °C. Despite that, De Pous et al. [34], during a series of short field trips to Sardinia that took place between 1999 and 2012, recorded just two specimens of *N. h. cetti*, both found basking during the daytime. Similarly, Lunghi and colleagues [28] observed 17 individuals active during the day between 10:08 and 16:42. Finally, the observations of the present study made by the authors, excluding individuals under shelters or found road-killed, all occurred in a daytime context. Since these surveys were taking place only in the daytime, the authors can’t rule out or quantify the extent of the nocturnal nature of *N. h. cetti*. It is plausible that, like the vast majority of the other members of the genus, the Sardinian grass snake alternates between a diurnal and nocturnal activity pattern according to the variation in biotic and abiotic factors. Adopting a nocturnal lifestyle, especially during summer, would allow *N. h. cetti* to decrease the risk of predation, loss of body fluids, and overheating, while increasing its foraging success (see [88]).

Various species of amphibians, which tend to be predominantly nocturnal, made up the vast proportion of the Sardinian grass snake’s diet [15,25,89]. Based on the limited data available, the dietary habits of *N. h. cetti* seem to differ from the habits of mainland grass snakes [89], where the diet of the latter includes a broader variety of prey items, such as fish and other terrestrial vertebrates (e.g., [89,90,91,92,93,94,95,96,97]). In fact, among 12 food items collected from specimens of *N. h. cetti*, Capula and colleagues [83] retrieved a single non-amphibian prey (Tyrrhenian wall lizard -*Podarcis tiliguerta*-). The rest of the prey items consisted of six adults and five tadpoles of the Sardinian tree frog (*Hyla sarda*). Interestingly, tree frogs (*Hyla* spp.) seem to be rarely preyed on by mainland adults *N. natrix* s.l., while they appear as part of the diet of juveniles (40–50 cm in length) [89,98]. Tyrrhenian painted frogs (*Discoglossus sardus*) do not appear in the few food items analysed, but it is probable to expect them in the diet of the subspecies, as also generically indicated in thematic books (e.g., [15,24,25,99]). Caudata amphibians can also be part of the diet of the Sardinian grass snake. *Natrix h. cetti* has been found within the same cave systems colonised by cave salamanders (*Speleomantes* spp.) [100]. Gené [58] reported a case of predation involving a brown cave salamander (*S. genei*) and a juvenile specimen of Sardinian grass snake. Furthermore, Lunghi and colleagues [85] recorded four specimens of *N. h. cetti* at various distances (15 m, 5.4 m, 75 m, 12 m) from the entrance of sub-horizontal caves, potentially foraging for *Speleomantes* spp. *Natrix h. cetti* has also been recorded within rivers inhabited by the Sardinian brook newt (*Euproctus platycephalus*) [101]. It is then likely that the Sardinian grass snake might represent a potential predator of this endangered amphibian [25]. Data regarding the diet of *N. h. cetti* are still quite scarce, so it’s possible that this endemic snake might feed up on a wider range of native and introduced species.

There is a tremendous knowledge gap regarding the reproductive biology of *N. h. cetti*. The taxon is oviparous, and its reproduction cycle likely resembles the one of the mainland *N. natrix* s.l. [25]. Natricidae snakes are known to form mating balls during early spring, where both male and female reproductive success seems to be positively correlated with body size [102]. At birth, hatchlings of *N. h. cetti* measure around 15 cm in length [27].

Data regarding predation upon Sardinian grass snakes are lacking. Despite that, generalist predators, such as the wild boar (*Sus scrofa*) and the hedgehog (*Erinaceus europaeus*), are known to feed on snakes as do snake-specialists such as the snake eagle (*Circaetus gallicus*) (e.g., [103,104,105,106]). These may represent potential predators of *N. h. cetti*.

*Natrix natrix* s.l. are aglyphous snakes [107] and the presence of oral glands secreting toxins is still under debate (See [108]). In this regard, studies addressing the Sardinian grass snake are totally lacking. With regards to the defence mechanisms towards humans, if disturbed, *N. h. cetti* may primarily try to flee and, in some cases (e.g., when manipulated and particularly stressed), it adopts behaviours such as hissing, death feigning, and emission of foul-smelling cloacal secretions (MRDN pers. obs.). Although very rare, cases of *N. helvetica* biting humans exist [108], but to date, they have never been reported for *N. h. cetti*.

#### 3.1.4. Conservation

Data providing the accurate population and conservation status of the Sardinian grass snake are still scarce, and this is further complicated by the intricate systematic history of this taxon.

This endemic subspecies was classified by the IUCN as “Critically Endangered” due to its restricted distribution and small population size [109]. Ozinga et al. [110] also listed the Sardinian grass snake as “Critically Endangered”. In the same way, Vanni and Cimmaruta [24] reported *Natrix helvetica cetti* to be “Critically Endangered” based on a population reduction of over 80% (A1ac), a fragmented distribution and occupancy decline (B1 + B2b (i, ii, iv, v)), and a declining effective population size of fewer than 250 individuals (C2a). Within a subsequent national assessment, Andreone et al. [111] listed *N. n. cetti* as “Vulnerable” to extinction due to its low area of occupancy (less than 2000 km^2^), its fragmented distribution, and its declining population (B2ab (ii, iv)). In fact, between 1985 and 2006, the occurrence of *N. h. cetti* declined by half, while later surveys that took place between 2008 and 2013 highlighted an occupancy decline of almost 90% [111]. Andreone and colleagues [111], based on previous taxonomic frameworks, considered the *N. h. cetti* populations of grass snakes from both Sardinia and Corsica, the latter now classified as *N. h. corsa* (see taxonomy insights in Section 3.1.1). As the Corsican population appears to harbour a large number of specimens, this could have affected the conservation status of *N. n. cetti* proposed by Andreone et al. [111]. In the last IUCN national assessment [26], *N. h cetti* is listed as “Endangered” due to its low and declining area of occupancy (<500 km^2^) (B2ab (ii)). The Sardinian grass snake is also currently listed under the EU Habitat Directive within Annex IV (92/43/CE) [24].

The exact threats responsible for the decline of *N. h. cetti* are yet to be defined. One of them seems to be represented by the ecological competition with the possibly introduced viperine snake (*N. maura*) [24,27]. Indeed, the causes behind the presence of *N. maura* in Sardinia have been debated for decades. Lanza [75] suggested either a potential anthropogenic introduction onto the island or a natural colonisation during the Messinian event when, due to the desiccation of the Mediterranean Sea, Sardinia and Corsica were connected to mainland Italy and Tunisia [112]. Based on morphological similarities between the Sardinian populations and the African one, Schätti [113] proposed a human-mediated introduction of the former population. In the same way, an African origin and an anthropogenic introduction were suggested by Poggesi and colleagues [114]. More recent genetic analyses highlighted the presence of shared mitochondrial haplotypes between the Sardinian populations and the Tunisian ones [115]. Further sampling confirmed the recent colonisation of Sardinia by *N. maura*, but this did not produce enough evidence to differentiate between a natural colonisation and a human-mediated one [18]. Regardless, the ecological impact of *N. maura* on *N. h. cetti* could be relevant. Although there is no evidence of correlation, in Corsica, where *N. maura* is not present, *N. n. corsa* is abundant and widely distributed [15,27], while in Sardinia, *N. h. cetti* is scarce and generally restricted to areas lacking *N. maura* [27]. On the island, the two species are characterised by overlapping diets [89], with *Hyla sarda* representing the main prey item for both *N. maura* and *N. h. cetti* [83,116]. Where the two snakes are sympatric, competition for resources seems to favour *N. maura*, affecting *N. h. cetti* in terms of abundance, body mass, and growth [27].

Habitat alteration represents another potential threat to the survival of this rare reptile. Within a Habitat Report [117], agricultural intensification has been highlighted as a “highly important pressure” for *N. h. cetti*. Similarly, Falcucci et al. [118] recorded an increase in heterogeneous agriculture, coupled with the abandonment of pastoral lands and an increase in montane forest cover. Furthermore, Puddu and colleagues [119] showed a significant decrease in agropastoral practices during the last decades, which has led to an exponential increase in the forest cover with detrimental consequences on a large portion of Sardinian biodiversity. Moreover, due to its touristic power, Sardinia has undergone a radical urbanisation process, especially in the proximity of coastal areas [120]. These factors are likely to reduce even more the already fragmented distributional range of *N. h. cetti*.

Another potential threat considered critical for the survival of the Sardinian grass snake is represented by its potentially reduced genetic diversity [117]. This could be associated with an increase in inbreeding, which could lead to a decline in fitness, reproductive potential, and even local extirpation [121,122]. The effects caused by a loss in genetic diversity are stronger in taxa characterised by small and isolated populations, where the levels of gene flow among populations are scarce [123,124]. Based on current distributional data, *N. h. cetti* persist in small and isolated populations, thus making this taxon particularly susceptible to the detrimental effects of genetic loss [24,25]. Despite that, no studies have so far investigated the genetic status of the Sardinian grass snakes. The authors believe that the use of cutting-edge genomic techniques should be implemented in order to assess the population structure and the genetic health of this cryptic and endemic reptile. This should be coupled with extensive field investigations aimed at better understanding the ecological habits and exact distribution of *N. h. cetti*. The implementation of such actions would allow to promptly individuate potential threats to the survival of the Sardinian grass snake and to establish effective conservation measures.

### 3.2. Distribution Update

**Figure 2:**
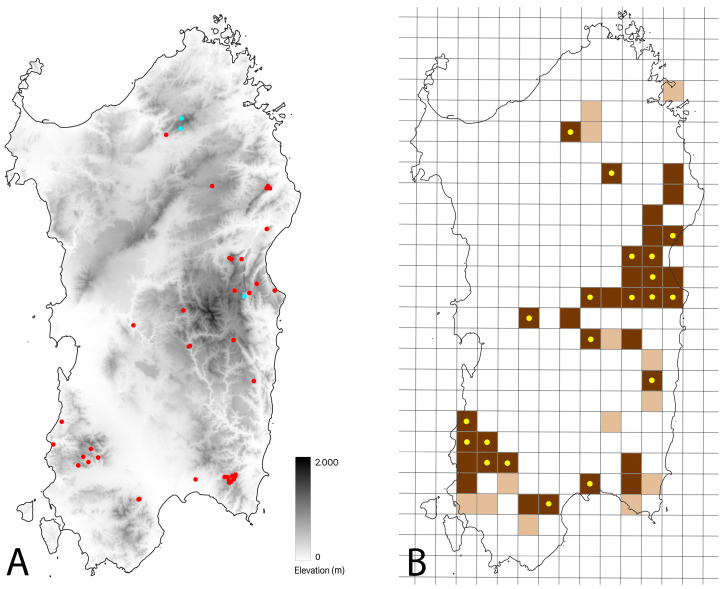
Map of Sardinia with the 64 observations of *Natrix helvetica cetti* provided with geographical indications: the red dots represent the post-2010 records, and the blue dots represent the pre-2010 ones (**A**). A 10 × 10 km UTM map of Sardinia with the updated distribution of *N. h. cetti*: the brown squares represent records from 2010 onwards, the beige squares represent pre-2010 records; brown squares with yellow dots represent new squares or reconfirmations of pre-2010 data with respect to what is indicated in [12] (**B**).

We collected a total of 66 verified observations of *Natrix helvetica cetti* (22 through personal surveys, 44 through collaborators and citizen science), carried out between 2004 and 2023, of which 92% (n = 61) were from 2010 onwards (Table S2). Among these, 97% (n = 64) were eligible for geographic mapping (Figure 2A) and were carried out in an altitude range between 20 e 1407 m a.s.l. (mean value = 586.8 m a.s.l.; see Figure S1). The collected data were merged with those presented by Corti et al. [12] in a 10 × 10 km UTM map: the 10 × 10 km cells affected by the presence of the taxon are 47, of which 13 are from pre-2010 and 34 are from 2010-onwards (Figure 2B).

*Natrix maura* occupies 131 UTM cells (of which 98 are known from Corti et al. [12] and 33 from personal observations and citizen science). From the overlay of the UTM distribution maps of *N. h. cetti* and *N. maura*, a co-presence emerges in 31 cells, equal to 66% of the total number of cells occupied by *N. h. cetti* and to 24% of the total number of cells occupied by *N. maura* (Figure S2).

According to Corti et al. [12], cave salamanders occupy a total of 61 UTM cells (*Speleomantes flavus* = 4, *S. supramontis* = 12; *S. imperialis* = 23; *S. sarrabusensis* = 5; *S. genei* = 17). From the overlay of the UTM distribution maps of *N. h. cetti* and *Speleomantes* spp., a co-presence emerges in 33 cells, equal to 70% of the total number of cells occupied by *N. h. cetti* and to 54% of the total number of cells occupied by *Speleomantes* spp. Specifically, *N. h. cetti* occurs in 3 out of 4 cells (75%) where *S. flavus* is present; in 10 out of 12 cells (83%) where *S. supramontis* is present; in 4 out of 23 cells (17%) where *S. imperialis* is present; in 3 out of 5 cells (60%) where *S. sarrabusensis* is present; and in 11 out of 17 cells (65%) where *S. genei* is present (Figure S3). Still in agreement with Corti et al. [12], the Sardinian brook newt occupies a total of 37 UTM cells. From the overlay of the UTM distribution maps of *N. h. cetti* and *Euproctus platycephalus*, a co-presence emerges in 18 cells, equal to 38% of the total number of cells occupied by *N. h. cetti* and to 48% of the total number of cells occupied by *E. platycephalus* (Figure S3). Ultimately, the co-presence between the Sardinian grass snake and at least one urodelan species occurs in 37 cells, equal to 79% of the total number of cells occupied by *N. h. cetti*.

### 3.3. Habitat Suitability Estimation

The models computed showed high discriminatory ability and low overfitting, with AUC values between 0.839 and 0.912 and OR10 values consistently below the 10% threshold (Table 2). Although all models led to robust statistical results, those including altitude as a predictor variable showed slightly higher AUC and lower OR. When included, altitude was, in fact, the most influential predictor for the distribution of *Natrix helvetica cetti*, followed by precipitation of the warmest quarter (bio18) and, in models using a reduced set of climatic variables, temperature seasonality (bio4). Based on the occurrences used for training the models, the distribution of the taxon showed a negative response to temperature variables (bio1–bio11, bio4 excl.) and a positive response to precipitation variables (bio12–bio19; bio15 excl.), whereas the most suitable land classes where the ones related to forested and herbaceous habitats (see Figure S4 for details).

**Figure 3:**
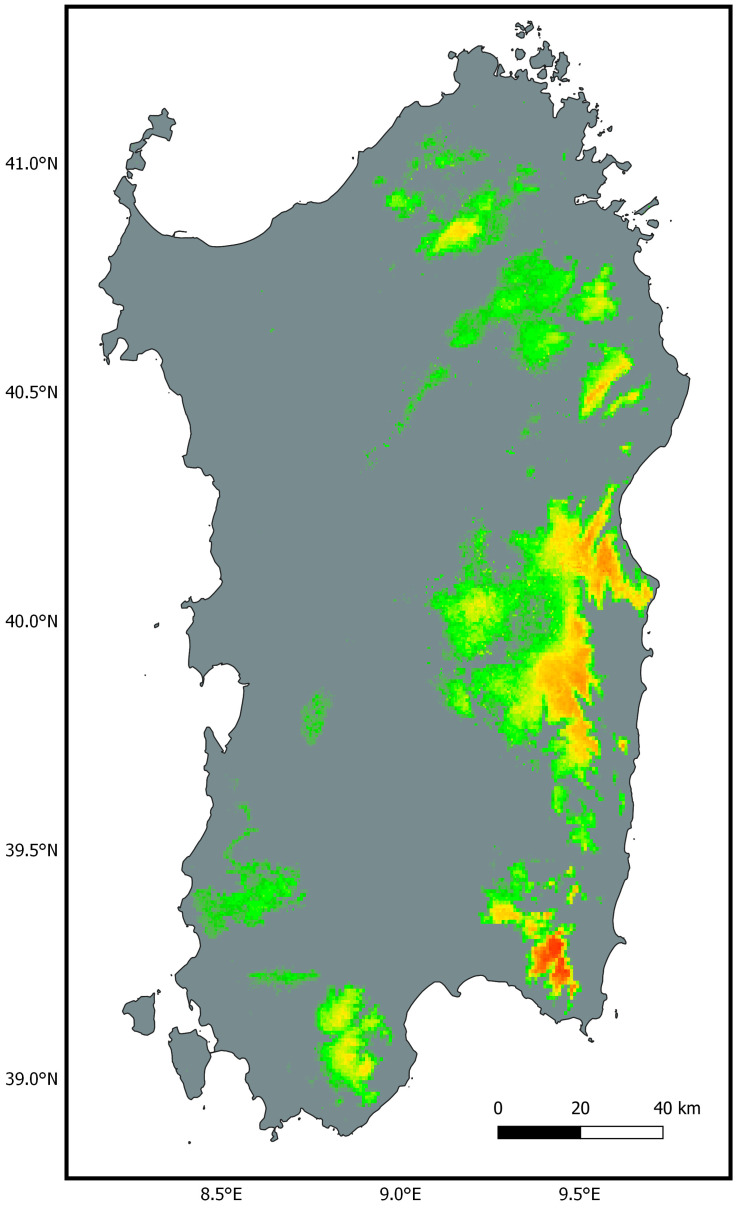
Predicted bioclimatic suitability for *Natrix helvetica cetti* in Sardinia. Warmer colours indicate higher suitability and grey areas fall below the minimum suitability threshold.

There was high consistency across the six models in the areas predicted as highly suitable for *N. h. cetti* in Sardinia (Figure S5). Models built upon the reduced set of bioclimatic variables showed a lower OR10 threshold for minimum suitability compared with models built with all bioclimatic variables (Table 2). Therefore, these models predicted a more widespread suitability for the taxon, although with arguably low suitability values (<0.15). These differences were minimised when adding altitude and then land cover as predictor variables to the models. The average model shows areas predicted as suitable for the taxon by all six models, using the mean OR10 (0.138) as the threshold for minimum suitability (Figure 3). This model predicted high suitability in the southeast of the island, specifically in the eastern part of the “Campidano di Cagliari” sub-region bordering the southern sector of the “Sarrabus-Gerrei”, and in the central-east of the island, especially in the “ Ogliastra” and secondly in the “Barbagia di Seulo”, in the southern portion of the “Barbagia di Nuoro” and in the eastern sectors of the “Barbagia di Belvì” and “Mandorlisai”. Further areas of high suitability are found in the northeast of the island, in the centre of the “Baronie” sub-region, and in the north of the island between the “Gallura” and “Monte Acuto” sub-regions. Finally, in the south-western sector of the island, two areas of medium-low suitability were highlighted in the south-east and in the north-west of the “Sulcis-Iglesiente”, with the latter case also encroaching on the southern sector of the “Monreale” (Figure S6). Figure S7 shows the predicted bioclimatic suitability areas compared to the updated distribution of *N. h. cetti*.

### 3.4. Dorsal Pattern Characterisation

**Figure 4:**
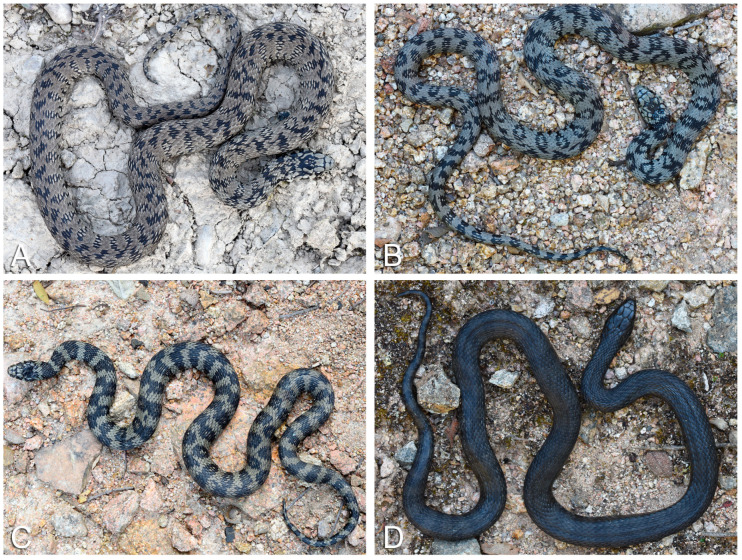
Examples of the four categories into which the dorsal patterns of *Natrix helvetica cetti* have been grouped: category I (**A**), category II (**B**), category III (**C**) and category IV (**D**).

On the basis of the prevalent trend of the dorsal motif observed in each considered snake, four different patterns have been identified for the Sardinian grass snake (Figure 4):

(I) Prevalence of thin lateral spots, sometimes light mottled, which do not reach the vertebral line; “third” row of spots along the vertebral line, tendentially offset from the lateral ones.

(II) Prevalence of lateral spots with an evident light central area, which reach the vertebral line, staggered or aligned (predominantly ocelli appearance).

(III) Tending to abundism: prevalence of thick lateral spots with reduced or absent light central area, which reach the vertebral line, staggered or aligned (predominantly bar-like appearance, or ocelli with thick border and narrow centre).

(IV) Melanic: uniformly dark dorsal colouration, with more or less visible dorsal pattern.

A total of 56 snakes were eligible for the pattern characterisation. Of these, 25% (n = 14) fell into cat. I, 46.43% (n = 26) in cat. II, 12.5% (n = 7) in cat. III, and 16.07% (n = 9) in cat. IV.

Out of 56 individuals with the dorsal pattern characterised, 54 had coordinates and have been computed in the 10 × 10 km UTM maps (Figure S8).

### 3.5. Principal Component Analysis

**Figure 5:**
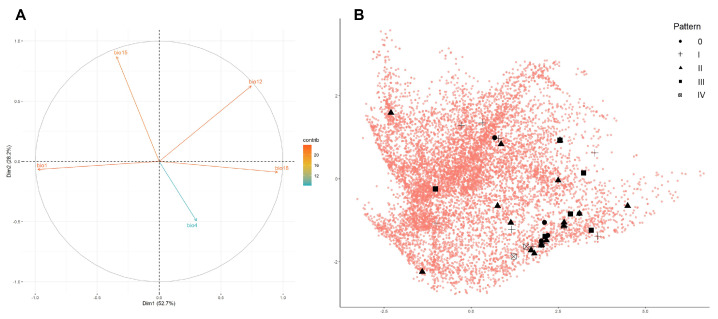
Principal component analysis of *Natrix helvetica cetti* in Sardinia. The first and second dimensions explain 52.7% and 28.2% of the variation observed in the data, respectively. (**A**) Bioclimatic variables used for defining the environmental space (warmer colours indicate higher variable contribution). (**B**) Occurrence records divided by dorsal pattern (black shapes) and represented in bidimensional environmental space against randomly sampled background points (red dots).

The result of the principal component analysis (PCA) of *Natrix helvetica cetti* shows an apparent differentiation of the bioclimatic niche of the taxon compared to the background environment (Figure 5). The first two PCA dimensions explain 52.7% (Dim1) and 28.2% (Dim2) of the variation observed in the data. Mean annual temperature (bio1) and precipitation of the warmest quarter (bio18) contribute mostly to the first dimension, whereas precipitation seasonality (bio15) and, to a lesser extent, temperature seasonality (bio4) explain most of the variation along the second dimension (Figure 5A). The PCA shows a certain degree of bioclimatic variation for the taxon, especially with regard to the first dimension. Individuals of *N. h. cetti* were found in areas characterised by lower temperatures, higher summer precipitations, lower precipitation seasonality, and higher temperature seasonality compared to the background environment. However, there is no apparent separation between the bioclimatic niche occupied by individuals with different dorsal patterns (Figure 5B).

## 4. Discussion

### 4.1. Natural History Review

*Natrix natrix* s.l. is a taxon with a complex taxonomic history, but various molecular studies carried out in recent years, including a recent genomic work, have helped to shed light on the phylogenetic relationships (e.g., [20,21,22,51,52,53,54,55]). This also applies to *N. helvetica cetti*, a taxon currently considered Sardinian endemic, but which could undergo updates regarding the inclusion of Corsican populations, currently kept separate due to the greater rarefaction of the Sardinian ones, which would need greater conservation attention [22,23].

From a morphological point of view, there are data for Sardinian populations mainly concerning morphometry, pholidosis, and even internal anatomy, while information on chromatic variability is still not very thorough (see [15,24,25,28,45,69,70,83]).

The ecological aspects of the Sardinian grass snake are still largely unknown and the little information in our possession comes from a limited number of studies, also concerning a small number of snakes. For example, more information on phenology, diet, and reproduction would be needed.

From a conservation point of view, *N. h. cetti* is a taxon adequately protected both by laws and categorisations in the global and national IUCN red lists. However, a greater effort in monitoring the populations would be useful in order to gain more precise information on their status. To date, there is no health and epidemiological information for Sardinian grass snakes. Recently, the European Food Safety Authority’s funder project “AMPHIDEB” aims to develop biologically-based models for environmental risk assessment and to assess the impact of chemicals and pathogenic fungi on amphibian and reptile populations (see [125]). In this context, individuals of *N. h. cetti* are being monitored for ophidiomycosis, an emerging infectious disease caused by an ascomycete fungus (see [126]) whose presence has recently been ascertained in Italy, involving the genus *Natrix* [127].

### 4.2. Distribution Update

Species geographic distribution is largely used during conservation assessments [128,129] and understanding the real extent of species’ geographic ranges is thus fundamental in order to reliably determine their conservation status and, if necessary, designate appropriate protected areas [7,130,131]. In the case of elusive and rare taxa occurring at low densities, the extent of their geographic range may be underestimated due to their low detectability and the occurrence of observer biases [132,133,134,135]. Here, combining personal survey effort, citizen science records and literature searches, we provided the most up-to-date geographic distribution of the Sardinian grass snake.

The Sardinian grass snake is characterised by a highly restricted and fragmented geographic range that mainly encompasses the mountainous areas of eastern and southern Sardinia [15,24,25]. Recent work by Corti and colleagues [12] aimed to update the distribution of *N. h. cetti*, as well as other Sardinian reptile and amphibian species, using distributional records extrapolated from the literature and surveys conducted between 2005 and July 2022. The authors reported the presence of *Natrix helvetica cetti* within 34 UTM cells, 14 (41.18%) of which were characterised by recent (from 2010 onwards) observational records, while the remaining 20 UTM cells (58.82%) included older (before 2010) observational records.

Our results highlighted the presence of the taxon within a total of 47 UTM cells (+13 UTM cells, an increase of +38.2%) (Figure 2B). The number of cells containing old unconfirmed observational records (before 2010) slightly differed from what was recorded by Corti et al. [12] (13 vs. 20, respectively). In addition, our dataset was characterised by more than a twofold increase in the number of cells harbouring recent (from 2010 onwards) observational data in comparison with what was highlighted by Corti and colleagues [12] (34 vs. 14, respectively). Notably, we were able to re-confirm the presence of *N. h. cetti* within 7 cells containing old observational data [12] and record the presence of the taxon in 13 previously overlooked cells, expanding in this way its known geographic occurrence quite significantly.

Natrix *h. cetti* is known in an altitudinal range between sea level and about 1000 m a.s.l., with a prevalent distribution in hilly and medium-mountainous areas [15,24,25], and the altitude record was 1029 m a.s.l. [28]. From the data available in the present study, the altitude limit rises to 1407 m a.s.l., while most of the records are within an altitude range between 440 and 730 m a.s.l. (Figure S1), confirming a mainly hilly and medium-mountainous distribution.

The discrepancy found between our results and those from previous studies may be due to a series of interacting factors. In the first place, there is no doubt that *N. h. cetti* occurs at low densities within its geographic range, making it extremely difficult to detect specimens even during tailored surveys ([31,85,134,136]; Di Nicola pers. obs.). Furthermore, the low detectability of the taxon may be also correlated with its still poorly understood activity pattern (see Section 3.1.3). In particular, the potential nocturnal nature of *N. h. cetti* may negatively affect the detectability and the encounter rate of the taxon [83]. The implementation of nocturnal surveys could provide conservation managers with new critical geographic data and contribute to exploring variations in the activity pattern of the Sardinian grass snakes throughout the active season, as has been conducted with other snake taxa (e.g., [137]). Finally, we believe that, aside from the authors’ personal survey effort, the richness of our dataset was partly associated with the exploitation of observational data obtained from citizen science (constituting 66.7% of the records). The exponential growth of these platforms during the last few years has in some cases allowed them to fill critical distributional knowledge gaps, even in the case of rare and rarely recorded species [138,139,140,141]. Accordingly, the authors acknowledge that citizen science platforms have an enormous potential for better understanding the real extent of the geographic distribution of *N. h. cetti*, especially taking into consideration the fact that Sardinia is becoming a popular destination for nature-oriented tourism [142,143].

### 4.3. Habitat Suitability Estimation

Understanding the role of bioclimatic variables in shaping rare and elusive species’ geographic range via the implementation of niche modelling methods is fundamental to better direct survey efforts towards unoccupied areas of potentially suitable habitat [144,145,146].

Our bioclimatic niche modelling analysis recovered the variable “altitude” (bio20) as the strongest predictor for the geographic distribution of *Natrix helvetica cetti.* This seems to agree with what is currently known about the ecology of the taxon. The Sardinian grass snakes seem in fact to mainly inhabit mid-elevation montane areas [25,28,34,86], as further highlighted by our altitudinal records (see Section 4.2). Nevertheless, specimens of Sardinian grass snakes have been found from the sea level up to over 1000 m a.s.l (see novel altitudinal record) [15,28].

When the variable “altitude” was excluded from the analysis, the “precipitation of the warmest quarter” (bio18) variable appeared as the most influential predictor for the distribution of the taxon (Figure S4). Taking into consideration that a vast proportion of Sardinia is characterised by dry conditions during the warmest quarter of the year, with levels of evaporations that surpass the precipitations [147], the tendency of *N. h. cetti* to inhabit areas with high rates of precipitations during the warmer period of the year may be correlated with the presence of water-dependent trophic resources (amphibians) [27].

More generally, the positive correlation with precipitation variables highlighted by our model may explain the predominantly eastern-restricted geographic distribution of *N. h. cetti* [15,24]. The eastern side of Sardinia is, in fact, characterised by higher rates of precipitation (mean annual precipitation and mean annual number of rainy days) compared to the western side of the island [148].

On the other hand, our model recovered a negative correlation with temperature bioclimatic variables. In particular, the distribution of *N. h. cetti* was negatively correlated with the mean annual temperature (bio1) and the temperature seasonality (bio4) variables. The latter negative correlation is likely linked to the high evaporation rate discussed above and consequently to the availability of trophic resources.

Finally, forested and herbaceous land cover categories were highlighted by our model as the most suitable for *N. h. cetti* (Figure S4). Individuals of Sardinian grass snakes are commonly found within forested areas [15,24,25] where high levels of humidity and soil moisture correlated with evapotranspiration patterns may influence amphibian abundance [149,150]. In contrast, herbaceous habitats seem to be poorly exploited by *N. h. cetti*. In fact, these habitats are highly subject to humidity loss and aridification [150,151]. Thus, we believe that the high response towards herbaceous land cover recovered by our model may be due to the resolution of the available land cover raster, which does not allow the distinction of microhabitats within larger herbaceous openings [152].

Indeed, the present results constitute a conservative, coarse-scale representation of the climatic suitability of the taxon on the island. Future studies should focus on gathering additional information regarding the local environment in which individuals are found. This would allow us to further refine the current models by implementing data on microhabitats (i.e., distance from small water courses, canopy cover, soil type, etc.), ecology (i.e., presence/absence of local prey/predators), and human impacts (i.e., land cover change, habitat disturbance, etc.) at a finer scale.

During the past decades, Sardinia has undergone a drastic decline in the rate of precipitation and a critical increase in temperature [153,154,155]. This has potentially reduced the suitable habitat for *N. h. cetti*, with consequent drastic population decline (red list). A further decrease in precipitation and a further increase in temperature are expected for Sardinia under future climatic scenarios [156]. Furthermore, future climatic variations are expected to negatively impact the distribution of the forested areas in Sardinia, thus further reducing the available habitat for *N. h. cetti* [156].

Climate change represents a serious threat to the persistence of *N. h. cetti*, and we argue that intensive monitoring is necessary to assess future distributional shifts and population contractions.

### 4.4. Geographic Distribution of Potential Competitors and Trophic Resources

In addition to climatic factors, species’ geographic distribution is limited by competition with other taxa and influenced by the presence of trophic resources.

Here we compared distributional data regarding *Natrix helvetica cetti* with those from a potential competitor, the viperine snake (*N. maura)*, and a potentially overlooked trophic resource, urodeles (*Speleomantes* spp. and *Euproctus platycephalus*).

Regarding the potential ecological competitor, our analyses, based on distributional data from Corti et al. [12], highlighted a striking overlap between the geographic range of *N. h. cetti* and the graphic range of *N. maura*. In fact, 31 UTM cells appeared to be shared between the two taxa (Figure S2), corresponding to 66% of the geographic range of the endangered Sardinian grass snake. It’s expected that niche partition and differences in habitat choice should reduce the competition by limiting sympatric occurrence between the endemic *N. h. cetti* and the possibly introduced *N. maura* [27]. Among these factors, altitude seems to play a crucial role, with the former taxon mainly inhabiting hilly and mid-mountainous areas and the latter species preferring humid areas from the sea level up to 660 m a.s.l. [15,24,25,157]. When the two taxa are found sympatrically, *N. maura* seems to outcompete *N. h. cetti*, with negative consequences on the fitness of the latter (see Section 3.1.4; [27]). Furthermore, changes in climatic conditions are likely to produce an altitudinal shift in the geographic range of *N. maura*, enhancing the sympatry between the two reptiles [158,159]. Under this scenario, the interspecific competition between the two taxa is likely to increase with potentially detrimental consequences on the conservation status of the already declining *N. h. cetti* [27]. While the use of UTM cells to investigate the overlapping range of the two snake species is limited by the spatial resolution of the data, our results still advocate for the need for surveys aimed at assessing shifts in the geographic range of *N. maura* and the consequent ecological and geographical response of the endemic *N. h. cetti.*

Additionally, we assessed the geographic overlap between the Sardinian grass snake and the six species of urodelan amphibians (five species of cave salamanders, *Speleomantes* spp., and the Sardinian brook newt, *Euproctus platycephalus*) endemic to the island (Figure S3). 

The results highlighted how a vast proportion of the geographic range of *N. h. cetti*, 33 out of the 47 UTM cells (70%), was interested in the presence of *Speleomantes* spp.

Unsurprisingly, a third of the communal UTM cells shared between the Sardinian grass snake and cave salamanders were interested in the presence of the brown cave salamander (*S. genei*) (11/33 UTM cells). The latter species is in fact characterised by one of the largest distributional ranges among all the Sardinian cave salamanders, in an altitude range between 8 and 600 m [12,15]. On the other hand, an unexpectedly high level of geographic overlap was found between *N. h. cetti* and the Supramonte cave salamander (*S. supramontis*) (10/33 UTM cells). This latter species is characterised by a highly restricted geographic range where it inhabits a wide variety of habitats from 106 to 1360 m a.s.l. [15], potentially suitable for the sympatric presence of *N. h. cetti*. The high level of geographic overlap between *N. h. cetti* and the five species of Sardinian cave salamanders may highlight the presence of an overlooked and crucial trophic resource for the former taxon. Individuals of Sardinian grass snake have been found within cave areas inhabited by *Speleomantes* spp. [85,100] and predatory episodes have been recorded [58]. Despite the limitation of UTM cells, we believe that further work aimed to investigate the tropic relationship between *N. h. cetti* and *Speleomantes* spp. may have drastic implications on the conservation of all the involved taxa.

The Sardinian brook newt occupies 38% of the cells where *N. h. cetti* is reported. This amphibian prefers aquatic environments with fresh and well oxygenated water, in an altitude range from 50 to 1,800 m a.s.l. (especially between 400 and 800 m a.s.l.) [15], contexts that are also suitable for *N. h. cetti*, for which the newt probably represent a prey, although not yet ascertained.

Anyway, the overlapping distribution between the Sardinian grass snake and the six species of urodelan ampbibians may be correlated with the biogeographic history of the island and the formation of significant biogeographic barriers. This peculiar distributional pattern of endemic taxa has been further highlighted by the recently published work of Corti and colleagues [12].

The highlighted overlap geographical pattern may represent a unique conservation opportunity. In fact, the institution of protected areas for the Sardinia grass snake is likely to have beneficial impacts on the endemic and threatened six urodelan species.

### 4.5. Geographic and Environmental Influence on Dorsal Pattern Variation

Snakes’ dorsal patterns have been shown to provide crucial antipredatory functions, such as crypsis, aposematism, and mimicry [160,161,162,163,164,165]. Furthermore, the amount of dark pigments within dorsal patterns has been demonstrated to influence an individual’s thermoregulatory abilities [166].

Consequently, an interplay of various selective forces is expected to shape intraspecific pattern variability, acting on a trade-off between predator avoidance and thermal benefits [158,167,168].

Here, we tentatively investigated the influence of distributional and environmental variables on the high dorsal pattern variation observed in the Sardinian grass snake. Nevertheless, taking into consideration the low encounter rate that characterises *Natrix helvetica cetti*, we potentially produced and analysed the largest dataset regarding the phenotypic variation in the Sardinian grass snake to date.

Firstly, no geographic correlation was highlighted by our analyses as the assessed dorsal pattern categories seem to be randomly distributed across the geographic range of the taxon (Figure S8). Furthermore, geographical overlapping, in terms of shared UTM cells, between individuals characterised by different dorsal patterns was frequently observed. The “ocellated” dorsal pattern (cat. II) appeared as the most common dorsal pattern category, characterising 46% of the assessed individuals across 13 UTM cells. This was followed by the “thinly banded” dorsal pattern (cat. I) (11 UTM cells). The historical presence of *Vipera aspis* complex members on the island until the late Pliocene [169,170] is highly unlikely to explain a potential mimicry function of these widely diffused dorsal patterns (e.g., [164]). On the other hand, it is possible that these dorsal pattern categories may be beneficial in terms of crypsis, promoting predator avoidance [162,171]. In this regard, the “banded” dorsal pattern (cat. III) may balance the trade-off between thermal advantages and predator detection [166]. Nevertheless, the lack of a defined bioclimatic niche (Figure 5) and the low geographic distribution of the latter dorsal pattern category (7 UTM cells), seem to not support this hypothesis.

Furthermore, our PCA analysis failed to find any evident distinction between the assessed dorsal pattern categories based on the evaluated bioclimatic variables (Figure 5B). It is possible that this phenomenon may be due to the active maintenance of polymorphism within populations [172,173,174]. Moreover, predation avoidance rather than climatic niche segregation may represent another potential explanation for our results [175].

Interestingly, despite the close geographic clustering observed between individuals characterised by the melanistic dorsal pattern (cat. IV) (Figure S8), our PCA analysis did not highlight any significant distinction between this and the other dorsal patterns based on the bioclimatic variables considered. Melanism in snakes has been largely associated with thermoregulatory benefits [176,177,178]. For example, Bury et al. [168] found a negative correlation between melanistic individuals of *N. natrix* and bioclimatic variables such as spring and winter temperatures. Nevertheless, harder-to-detect selective forces have been shown to influence the intrapopulation abundance of melanistic snakes [179]. Data density represents a crucial factor for the reliability of PCA analysis [180]. The scarce number of melanistic individuals within our dataset may explain the inconclusive results of our PCA analysis. Despite this, taking into consideration the restricted geographic area where the vast majority of the melanistic *N. h. cetti* occur, it is possible that other mechanisms rather than the influence of bioclimatic factors may be responsible for the abundance of melanistic individuals (e.g., [176]).

## 5. Conclusions

*Natrix helvetica cetti* is an insular endemic grass snake subspecies for which many ecological aspects, as well as the actual distribution, are still poorly understood. The low detectability of the taxon and the scarcity of targeted field surveys probably led to this lack of information.

Exploiting some herpetological surveys by two of the authors and citizen science records, the present work had the main purpose of providing updated distribution data for the taxon, as well as giving information on habitat suitability and on potential distribution through bioclimatic niche modelling. Second, a preliminary characterization of the dorsal pattern was made with an attempt to explore the pattern of geographic segregation and to evaluate the influence of bioclimatic variables.

The fact that much of the data comes from citizen science has limited its potential for use. For example, it was not possible to take advantage of photos taken in standardized conditions or to know the morphometries, sex, microhabitat, and precise context of discovery for all the animals. Therefore, it will be important to carry out further field surveys aimed at acquiring this data and at implementing the ecological knowledge of the species, also carrying out investigations on the diet, of which little is known, and on the reproductive biology, for which information is almost totally lacking. The non-invasive acquisition of a set of environmental parameters will also be important (see [181]), such as body temperature, environmental temperature, illumination, and ultraviolet flux at the place of detection of an individual, as well as carrying out parasitological investigations (in addition to those underway for ophidiomycosis; see Section 4.1). Other ecological aspects, the knowledge of which will be fundamental for planning conservation strategies for the Sardinian grass snake, are the sheltering behaviour, the seasonal activity, the habitat trophism, the predation pressure, and the possible human mortality rate. By obtaining all the above information, it would be possible to provide targeted suggestions on the maintenance of populations, e.g., supporting the food supply and competition, controlling the competition with allochthonous and synanthropic animals, reducing the influence of nuisance factors, creating shelters and, although it is probably a limited phenomenon given the elusiveness and distribution of the species, preventing death on the roads.

As highlighted by our mapping results, the Sardinian grass snake seems to be characterised by a highly fragmented and limited geographic distribution. Unfortunately, the lack of historical data on the past distribution and abundance of this snake may have led to underestimating its current decline, and therefore, its current conservation status. Furthermore, this phenomenon may be even worsened by the almost complete lack of quantitative data regarding patterns of intraspecific competition with the possibly introduced viperine snake.

Therefore, we believe that an integrative approach is needed in order to assess the real extent of the decline of *Natrix helvetica cetti* and, if necessary, to put in place effective conservation measures.

## Figures and Tables

**Table 1:** List of reptiles of Sardinia. The subspecific framework is in agreement with [16].

Species and Subspecies	Notes
*Emys orbicularis* (Linnaeus 1758) *Emys orbicularis galloitalica* Fritz, 1995	
*Trachemys scripta* (Thunberg in Schoepff, 1792) *Trachemys scripta elegans* (Wied 1838) *Trachemys scripta scripta* (Thunberg in Schoepff 1792)	Recently introduced allochthonous species.
*Testudo hermanni* Gmelin 1789 *Testudo hermanni hermanni* Gmelin 1789	
*Testudo graeca* Linnaeus 1758 *Testudo graeca nabeulensis* (Highfield 1990)	
*Testudo marginata* Schoepff, 1792 *Testudo marginata marginata* Schoepff, 1792	
*Caretta caretta* (Linnaeus 1758) *Caretta caretta caretta* (Linnaeus 1758)	
*Euleptes europaea* (Gené, 1839) *Euleptes europaea europaea* (Gené 1839)	Species endemic to the Central-Western-Mediterranean.
*Hemidactylus turcicus* (Linnaeus, 1758) *Hemidactylus turcicus turcicus* (Linnaeus 1758)	
*Tarentola mauritanica* (Linnaeus, 1758) *Tarentola mauritanica mauritanica* (Linnaeus 1758)	
*Algyroides fitzingeri* (Wiegmann, 1834) *Algyroides fitzingeri fitzingeri* (Wiegmann 1834)	Species endemic to Sardinia and Corsica.
*Archaeolacerta bedriagae* (Camerano, 1885) *Archaeolacerta bedriagae bedriagae* (Camerano 1885)	Species endemic to Sardinia and Corsica.
*Podarcis siculus* (Rafinesque, 1810) *Podarcis siculus siculus* (Rafinesque-Schmaltz 1810)	
*Podarcis tiliguerta* (Gmelin, 1789) *Podarcis tiliguerta tiliguerta* (Gmelin, 1789)	Species endemic to Sardinia and Corsica. The validity of the subspecies *P. t. ranzii* and *P. t. toro* is not currently confirmed.
*Chalcides chalcides* (Linnaeus, 1758) *Chalcides chalcides vittatus* (Leuckart, 1828)	
*Chalcides ocellatus* (Forskål, 1775) *Chalcides ocellatus tiligugu* (Gmelin 1789)	
*Hemorrhois hippocrepis* (Linnaeus, 1758) *Hemorrhois hippocrepis hippocrepis* (Linnaeus 1758)	
Hierophis viridiflavus (Lacépède, 1789) *Hierophis viridiflavus viridiflavus* (Lacépède, 1789)	
*Natrix helvetica* (Lacépède 1789) *Natrix helvetica cetti* Gené, 1839	Subspecies provisionally considered endemic to Sardinia (see Section 3.1.1).
*Natrix maura* (Linnaeus, 1758) *Natrix maura* (Linnaeus 1758)	

**Table 2:** Statistical results of the six bioclimatic suitability models computed in MaxEnt. Mean (±SD) area under curve (AUC) values higher than 0.75 indicate good model quality, with values over 0.9 indicating excellent predictive performance (Fielding and Bell, 1997 [38]). A mean (±SD) 10th percentile training omission rate (OR10) lower than the predicted 0.1 denotes no overfitting (Boria et al., 2014 [39]). The OR10 threshold was used to set the minimum suitability values for the relative model (Figure S5).

Model	Variables	#Training Samples	#Test Samples	Test AUC	OR10 Threshold	OR10 Omission
1	Climate	28	12	0.888 ± 0.042	0.165	0.071
2	Climate Altitude	28	12	0.912 ± 0.036	0.164	0.068
3	Climate Altitude Land cover	28	12	0.892 ± 0.041	0.141	0.071
4	Climate, reduced	28	12	0.839 ± 0.051	0.101	0.071
5	Climate, reduced Altitude	28	12	0.889 ± 0.042	0.130	0.064
6	Climate, reduced Altitude Land cover	28	12	0.879 ± 0.050	0.127	0.068

## Data Availability

The data presented in this study are available on request from the corresponding author. The data (i.e., exact find coordinates) are not publicly available due to conservation reasons for the taxon involved.

## Supplementary Materials

The following supporting information can be downloaded at: https://www.mdpi.com/article/10.3390/life13091867/s1, Table S1: Valid *Natrix natrix* complex subspecies, and their approximate distribution ranges, according to Fritz and Schmidtler (2020) and Fritz and Ihlow (2022); Table S2: List of verified observations of *Natrix helvetica cetti* considered in the study; Figure S1: Altitudinal plot of the 64 *Natrix helvetica cetti* observations provided with geographic information; Figure S2: 10 × 10 km UTM map of Sardinia with the distribution of *Natrix helvetica cetti* (brown squares: post-2010 records; beige squares: pre-2010 records) and *N. maura* (yellow circles); Figure S3: 10 × 10 km UTM map of Sardinia with the distribution of *Natrix helvetica cetti* (brown squares: post-2010 records; beige squares: pre-2010 records) and urodeles (blue dots: *Speleomantes flavus*; red dots: *S. supramontis*; green dots: *S. imperialis*; yellow dots: *S. sarrabusensis*; violet dots: *S. genei*; yellow circles: *Euproctus platycephalus*).; Figure S4: Response curves showing the change in bioclimatic suitability for *Natrix helvetica cetti* (*y* axes) in response to variation in the predictor variables (*x* axes) used in the models; Figure S5: Predicted bioclimatic suitability for *Natrix helvetica cetti* in Sardinia based on six model combinations; Figure S6: Predicted bioclimatic suitability for *Natrix helvetica cetti* in Sardinia (see Figure 3 in the main document) superimposed on the map of Sardinian sub-region; Figure S7: Predicted bioclimatic suitability for *Natrix helvetica cetti* in Sardinia (see Figure 3 in the main document) superimposed on the 10 × 10 km UTM map with the updated distribution of the taxon (see Figure 2A in the main document); Figure S8: Distribution of the four different *Natrix helvetica cetti* dorsal patterns in the 10 × 10 km UTM map of Sardinia.
