# Supplementary material for: The Endangered Sardinian Grass Snake: Distribution Update, Bioclimatic Niche Modelling, Dorsal Pattern Characterisation, and Literature Review
Source: Life (Basel). 2023 Sep 4;13(9):1867. doi: 10.3390/life13091867 (PMC10533143; doi:10.3390/life13091867)
Supplement: Supplementary file 1 [file life-13-01867-s001.zip › life-2557242-supplementary.pdf]

The Endangered Sardinian Grass Snake: Distribution Update, Bioclimatic Niche Modelling, Dorsal Pattern Characterisation, and Literature Review

Matteo Riccardo Di Nicola[1,2,3,*], Andrea Vittorio Pozzi[4], Sergio Mezzadri[5], Francesco Paolo Faraone[6], Giorgio Russo[7], Jean Lou M. C. Dorne[8] and Gianmarco Minuti[9]

[1] Faculty of Veterinary Medicine, Department of Pathobiology, Pharmacology and Zoological Medicine, Wildlife Health Ghent, Ghent University, 9820 Merelbeke, Belgium
[2] Unit of Dermatology and Cosmetology, IRCCS San Raffaele Hospital, Via Olgettina 60, 20132 Milan, Italy
[3] Asociación Herpetológica Española, Apartado de correos 191, 28911 Leganés, Spain
[4] Molecular Ecology and Evolution Group, School of Natural Sciences, Bangor University, Bangor LL57 2UW, UK
[5] Independent Researcher, Via Palmerio, 29121 Piacenza, Italy
[6] Dipartimento Scienze e Tecnologie Biologiche, Chimiche e Farmaceutiche, University of Palermo, Via Archirafi 18, 90123 Palermo, Italy
[7] VIB-VUB Center for Structural Biology, Vrije Universiteit Brussel, 1090 Brussels, Belgium
[8] Methodology and Scientific Support Unit, European Food Safety Authority (EFSA), Via Carlo Magno 1A, Parma 43126, Italy
[9] Department of Biology, Ecology & Biodiversity Research Unit, Vrije Universiteit Brussels, 1090Brussels, Belgium

* Correspondence: dinicola.matteo@hsr.it

SUPPLEMENTARY MATERIALS

Subspecies	Distribution
Natrix astreptophora astreptophora (Seoane, 1884)	Iberian Peninsula and Occitany (France)
Natrix astreptophora algerica (Hecht, 1930)	Mediterranean region of central and eastern Algeria, Tunisia.
Natrix helvetica helvetica (Lacepède, 1789)	Western Europe, from the Pyrenees to the Rhine region, Great Britain.
Natrix helvetica sicula (Cuvier, 1829)	Sicily and mainland Italy, across the Alps to southernmost Bavaria (Germany).
Natrix helvetica cetti Gené, 1839	Sardinia (Italy)
Natrix helvetica corsa (Hecht, 1930)	Corsica (France)
Natrix natrix natrix (Linnaeus, 1758)	Central Europe and Scandinavia
Natrix natrix vulgaris Laurenti, 1768	Southern Central Europe and the northern Balkans
Natrix natrix moreotica (Bedriaga, 1882)	Southern Balkans, western Anatolia and Cyprus
Natrix natrix scutata (Pallas, 1771)	From eastern Poland and central Anatolia to Lake Baikal

Table S1. Valid *Natrix natrix* complex subspecies and their approximate distribution ranges, according to Fritz and Schmidtler (2020) and Fritz and Ihlow (2022).

Individual	Year	Protected coordinates	Altitude (m a.s.l.)	Pattern category
1	2004	40.***, 9.***	537	IV
2	2015	39.***, 9.***	584	IV
3	2019	39.***, 9.***	691	II
4	2022	39.***, 8.***	396	I
5	2022	39.***, 9.***	884	II
6	2022	39.***, 9.***	773	IV
7	2022	39.***, 9.***	784	II
8	2016	40.***, 9.***	798	II
9	2011	39.***, 8.***	417	N/A
10	2022	40.***, 9.***	920	III
11	2017	39.***, 8.***	451	I
12	2020	40.***, 9.***	726	I
13	2019	39.***, 9.***	876	N/A
14	2017	39.***, 9.***	703	II
15	2017	39.***, 9.***	689	II
16	2017	39.***, 9.***	516	II
17	2017	39.***, 9.***	525	II
18	2017	39.***, 9.***	577	N/A
19	2021	39.***, 9.***	727	IV
20	2021	39.***, 9.***	561	IV
21	2021	39.***, 9.***	656	II
22	2021	39.***, 9.***	522	IV
23	2022	39.***, 8.***	530	I
24	2018	40.***, 9.***	1407	II
25	2008	40.***, 9.***	642	II
26	2008	40.***, 9.***	927	I
27	2009	40.***, 9.***	649	N/A
28	2014	40.***, 9.***	985	II
29	2016	40.***, 9.***	710	II
30	2016	40.***, 9.***	842	N/A
31	2018	40.***, 9.***	983	I
32	2021	39.***, 9.***	333	II
33	2019	39.***, 9.***	553	II
34	2022	39.***, 8.***	123	III
35	2020	39.***, 9.***	543	II
36	2011	39.***, 9.***	672	N/A
37	2011	39.***, 9.***	693	N/A
38	2018	39.***, 9.***	73	II
39	2019	39.***, 9.***	555	II
40	2018	39.***, 9.***	502	I
41	2018	39.***, 9.***	927	III
42	2021	39.***, 8.***	249	I
43	2021	40.***, 9.***	940	I
44	2002	40.***, 9.***	649	III
45	2014	40.***, 9.***	440	II
46	2014	40.***, 9.***	440	N/A

47	2019	40.***, 9.***	553	I
48	2020	39.***, 8.***	118	N/A
49	2022	39.***, 9.***	553	II
50	2013	N/A	N/A	II
51	2012	N/A	N/A	IV
52	2011	39.***, 9.***	674	III
53	2018	39.***, 9.***	569	II
54	2018	39.***, 9.***	569	I
55	2018	39.***, 9.***	569	IV
56	2019	39.***, 9.***	569	IV
57	2018	39.***, 9.***	569	III
58	2020	40.***, 9.***	925	III
59	2018	39.***, 8.***	34	II
60	2019	40.***, 9.***	244	I
61	2019	40.***, 9.***	244	I
62	2018	39.***, 9.***	343	II
63	2021	40.***, 9.***	405	I
64	2023	39.***, 8.***	20	N/A
65	2023	39.***, 9.***	865	II
66	2019	39.***, 8.***	55	II

Table S2. List of verified observations of *Natrix helvetica cetti* considered in the study.

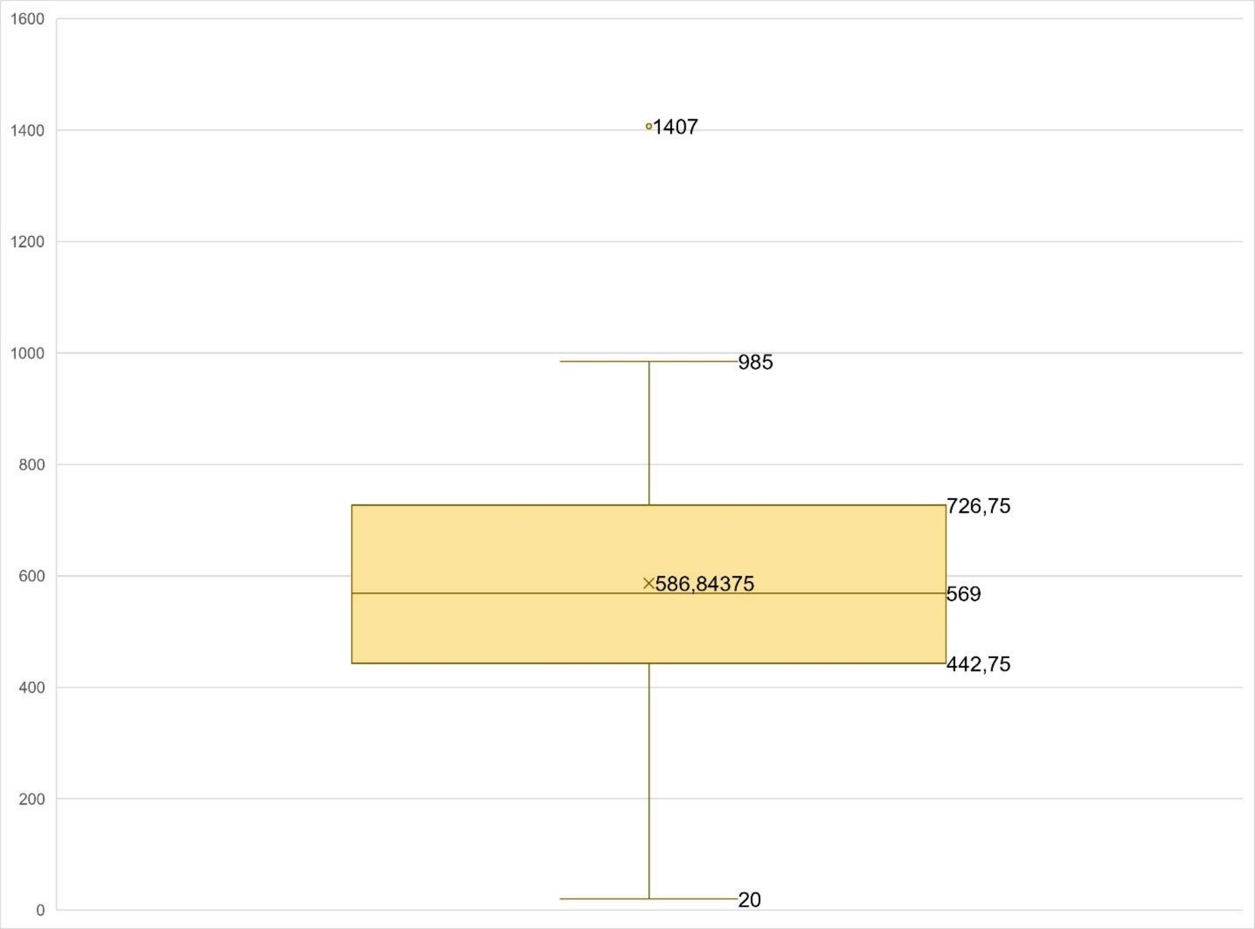

Figure S1. Altitudinal plot of the 64 *Natrix helvetica cetti* observations provided with geographic information.

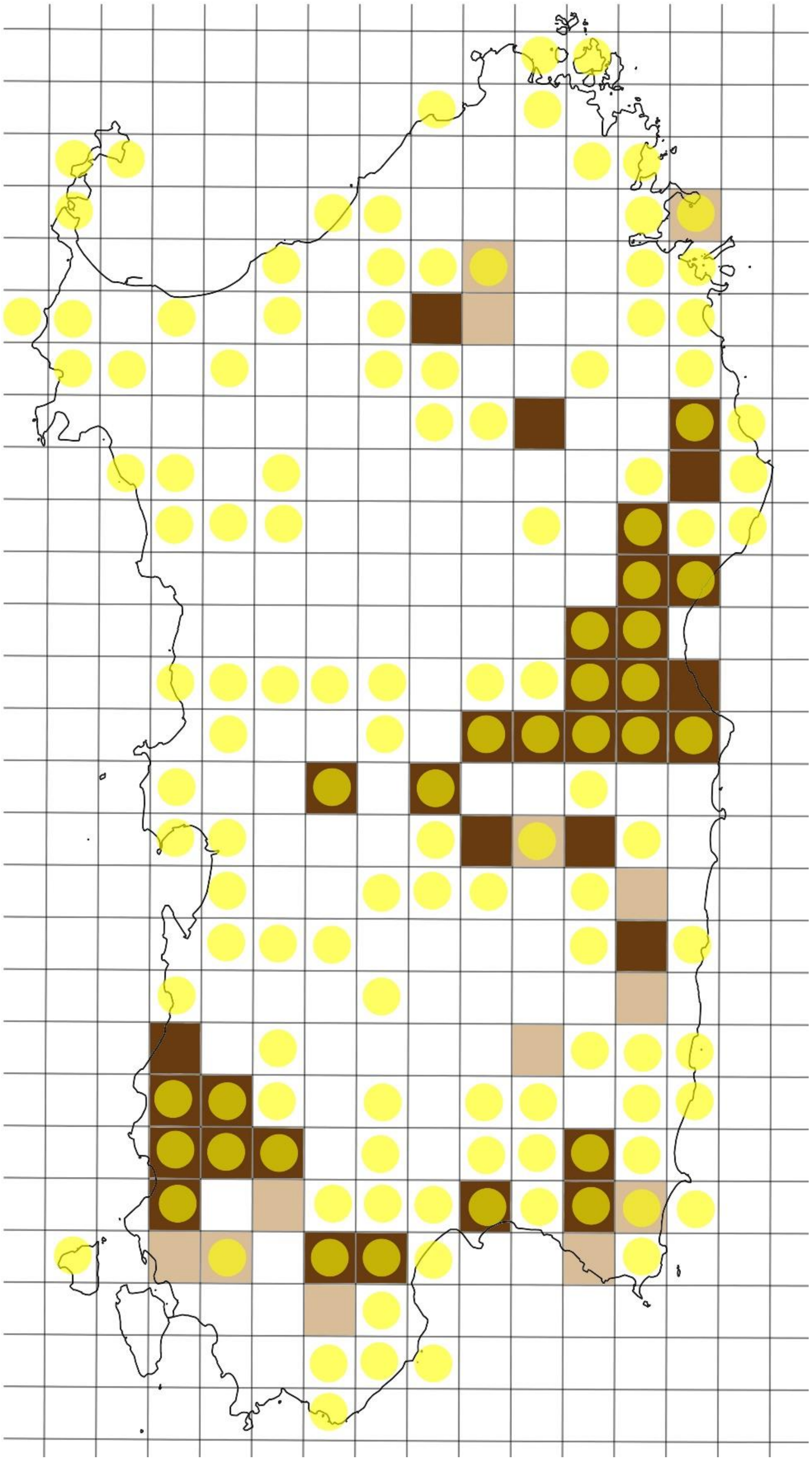

Figure S2. 10x10 km UTM map of Sardinia with the distribution of *Natrix helvetica cetti* (brown squares: post-2010 records; beige squares: pre-2010 records) and *N. maura* (yellow circles).

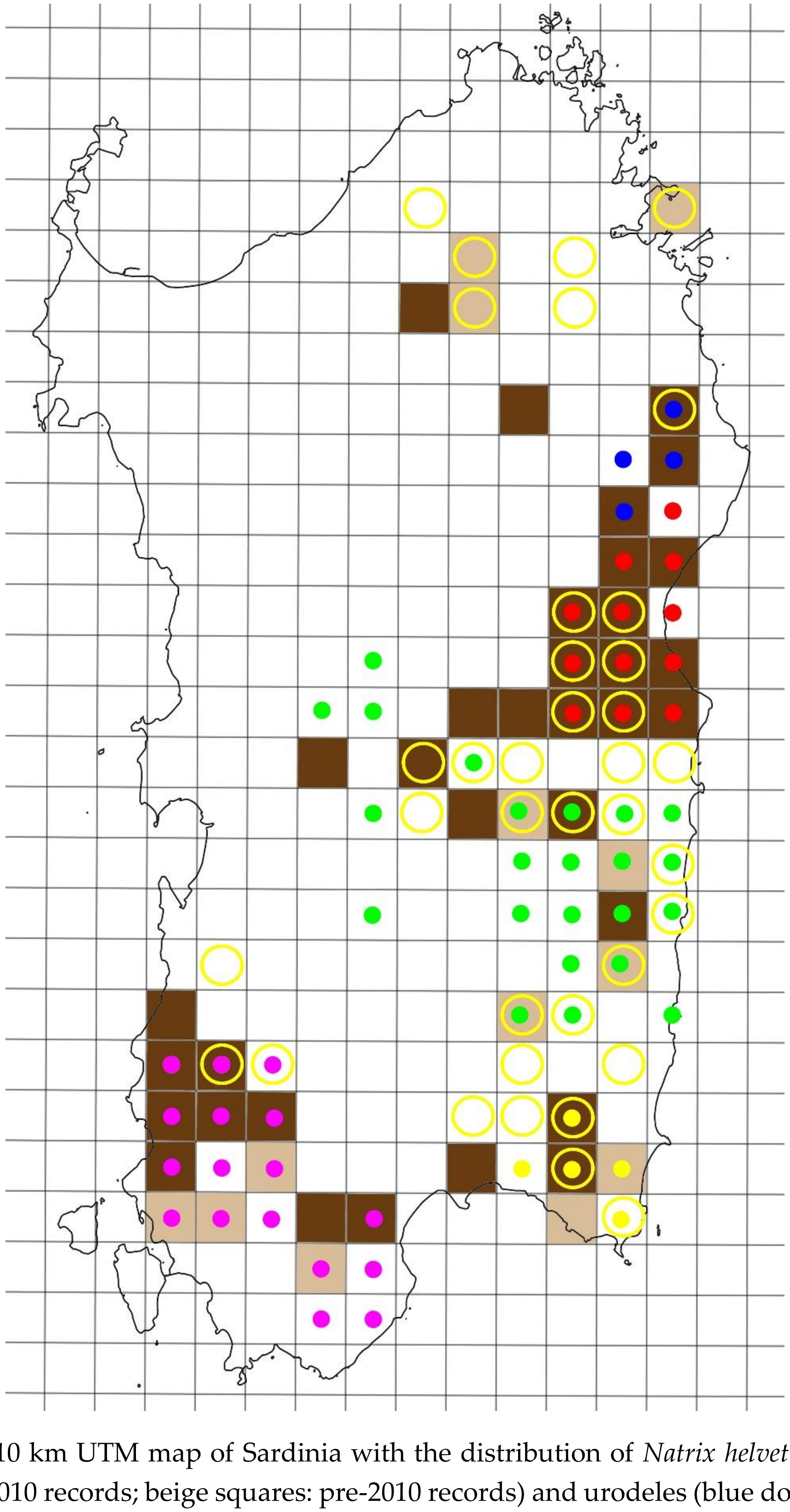

Figure S3. 10x10 km UTM map of Sardinia with the distribution of *Natrix helvetica cetti* (brown squares: post-2010 records; beige squares: pre-2010 records) and urodeles (blue dots: *Speleomantes flavus*; red dots: *S. supramontis*; green dots: *S. imperialis*; yellow dots: *S. sarrabusensis*; violet dots: *S. genei*; yellow circles: *Euproctus platycephalus*).

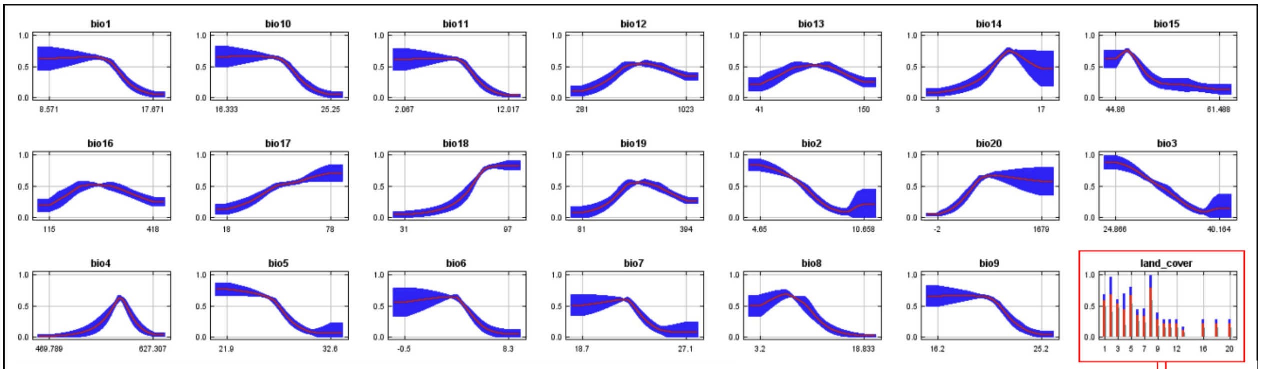

Code	Class Name	Code	Class Name
1	Broadleaf evergreen forest	11	Cropland
2	Broadleaf deciduous forest	12	Paddy field
3	Needleleaf evergreen forest	13	Other vegetation mosaic
4	Needleleaf deciduous forest	14	Mangroves
5	Mixed forest	15	Wetland
6	Tree open	16	Bare area (gravel, rock)
7	Shrubland	17	Bare area (sand)
8	Herbaceous	18	Urban area
9	Sparse trees/shrubs	19	Snow / ice
10	Sparse vegetation	20	Water bodies

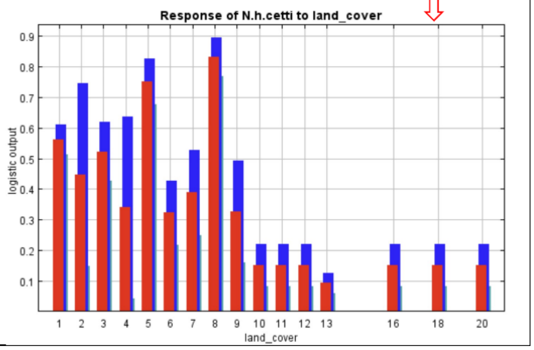

Figure S4. Response curves showing the change in bioclimatic suitability for *Natrix helvetica cetti* (y axes) in response to variation in the predictor variables (x axes) used in the models. Similar response curves denote highly correlated variables. Codes are as follows: bio1 = mean annual temperature; bio2 = mean diurnal temperature range; bio3 = isothermality; bio4 = temperature seasonality; bio5 = maximum temperature of the warmest month; bio6 = minimum temperature of the coldest month; bio7 = temperature annual range; bio8 = mean temperature of the wettest quarter; bio9 = mean temperature of the driest quarter; bio10 = mean temperature of the warmest quarter; bio11 = mean temperature of the coldest quarter; bio12 = annual precipitation; bio13 = precipitation of the wettest month; bio14 = precipitation of the driest month; bio15 = precipitation seasonality; bio16 = precipitation of the wettest quarter; bio17 = precipitation of the driest quarter; bio18 = precipitation of the warmest quarter; bio19 = precipitation of the coldest quarter; bio20: altitude (https://www.worldclim.org/data/bioclim.html). The inset highlights the response to the variable land cover, with relative legend (https://globalmaps.github.io/glcnmo.html).

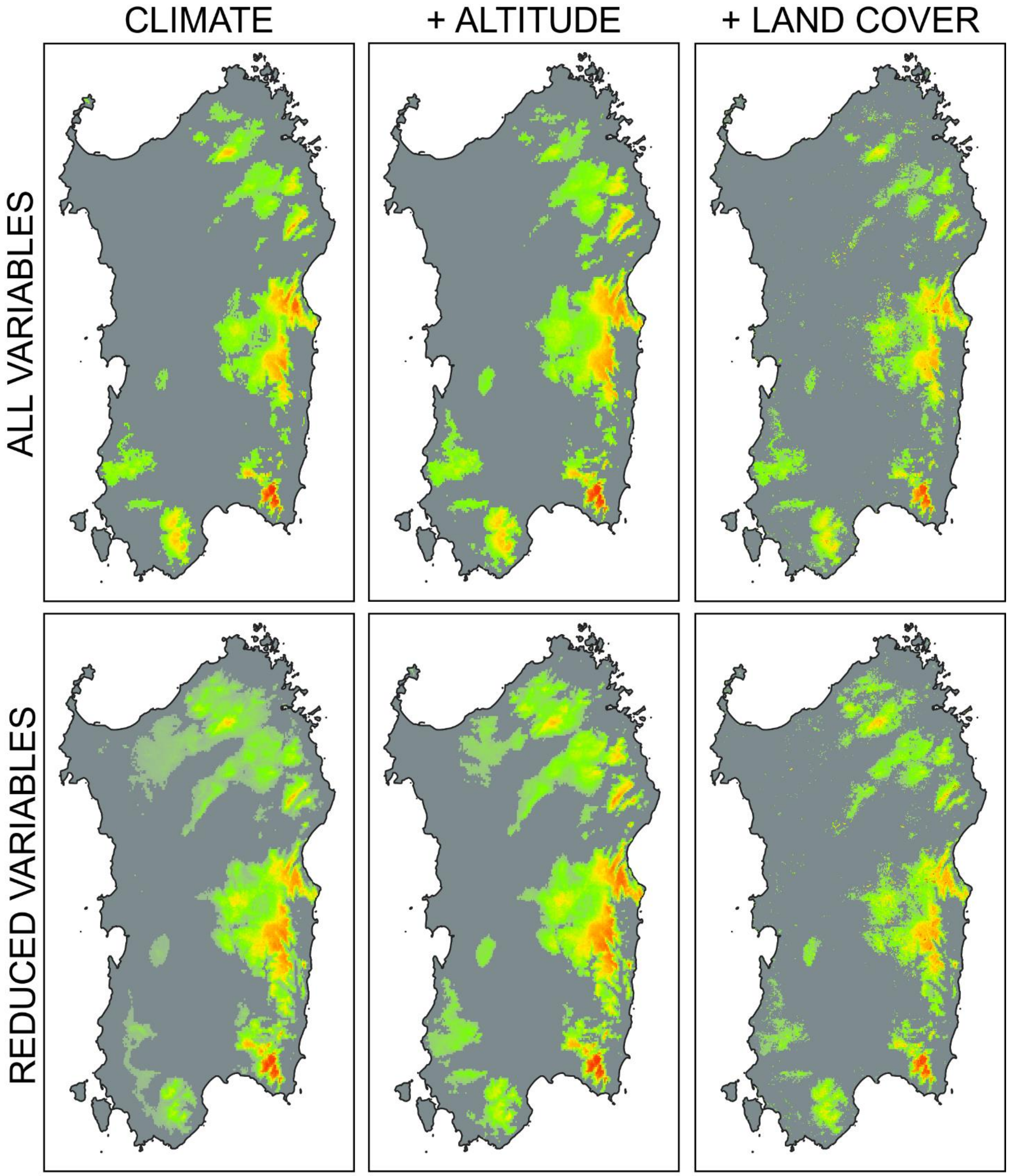

Figure S5. Predicted bioclimatic suitability for *Natrix helvetica cetti* in Sardinia based on six model combinations. Models were trained either on all 19 bioclimatic variables obtained from WorldClim 2.1 (Fick & Hijmans, 2017) or on a reduced set of variables selected to minimize multicollinearity between predictors (see section 2.3 for details). Altitude and land cover were subsequently added in a step-wise fashion to observe their effect on model output. Warmer colours indicate higher bioclimatic suitability; grey areas fall below the minimum suitability threshold.

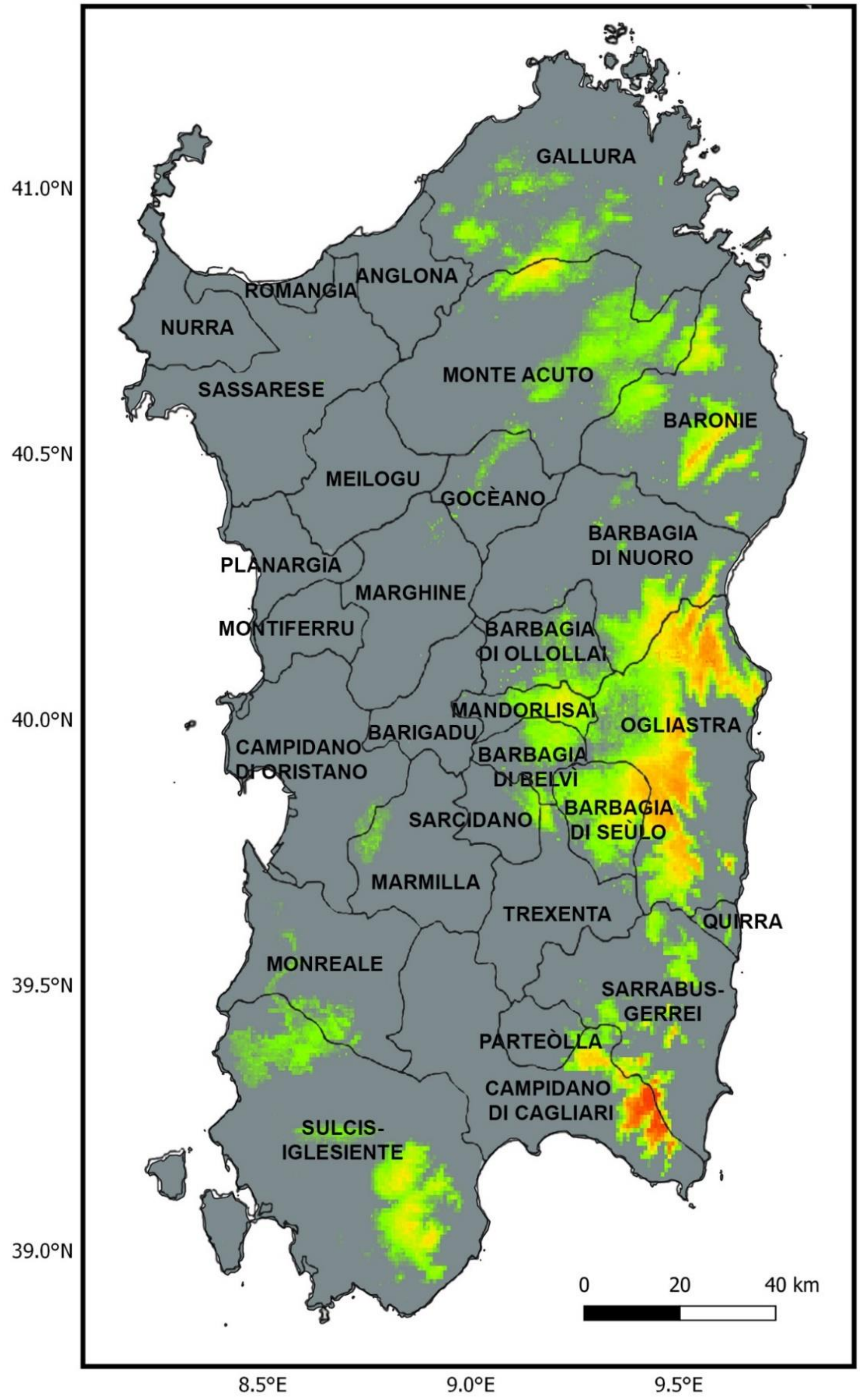

Figure S6. Predicted bioclimatic suitability for *Natrix helvetica cetti* in Sardinia (see Figure 3 in the main document) superimposed on the map of Sardinian sub-regions.

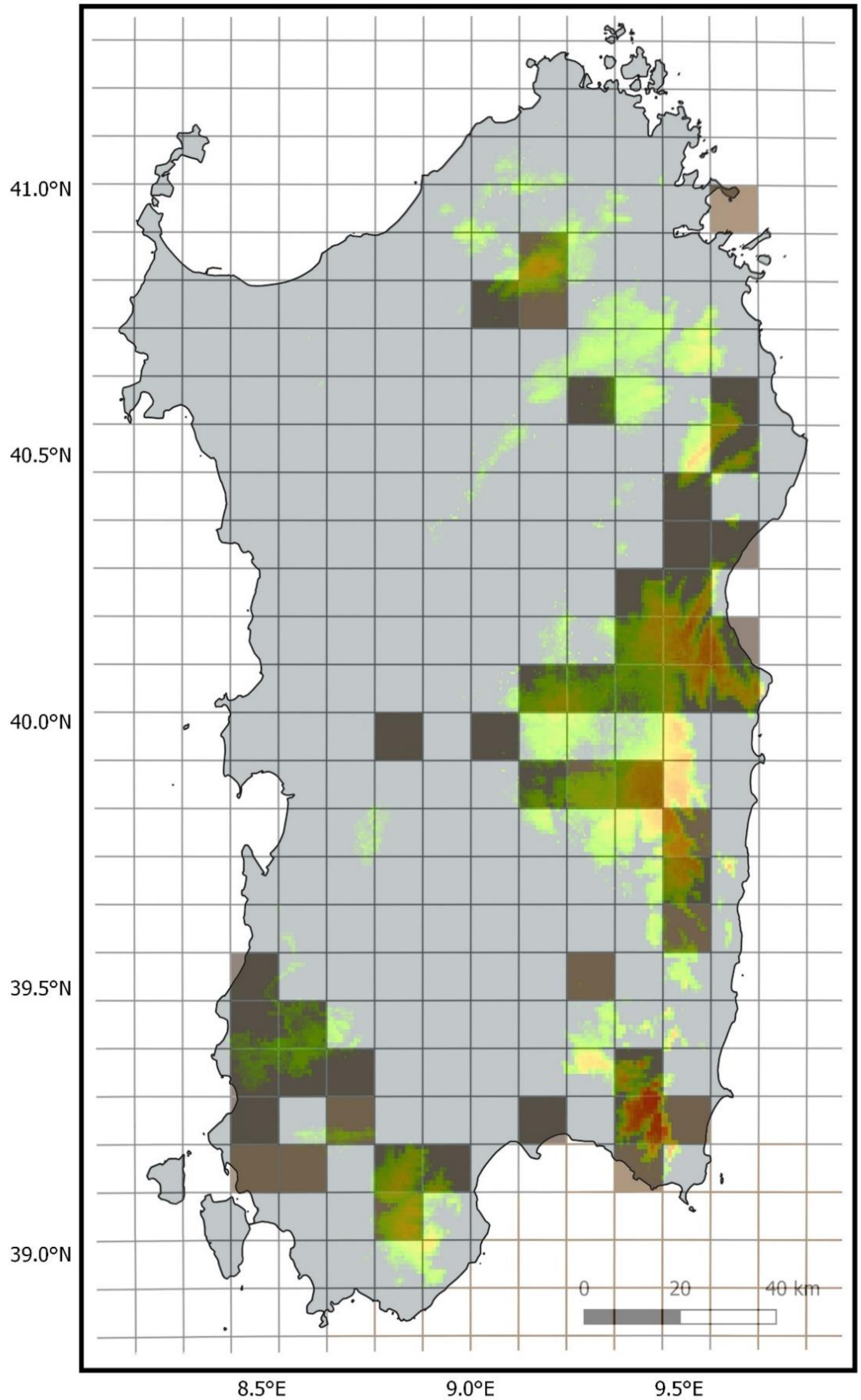

Figure S7. Predicted bioclimatic suitability for *Natrix helvetica cetti* in Sardinia (see Figure 3 in the main document) superimposed on the 10x10 km UTM map with the updated distribution of the taxon (see Figure 2A in the main document).

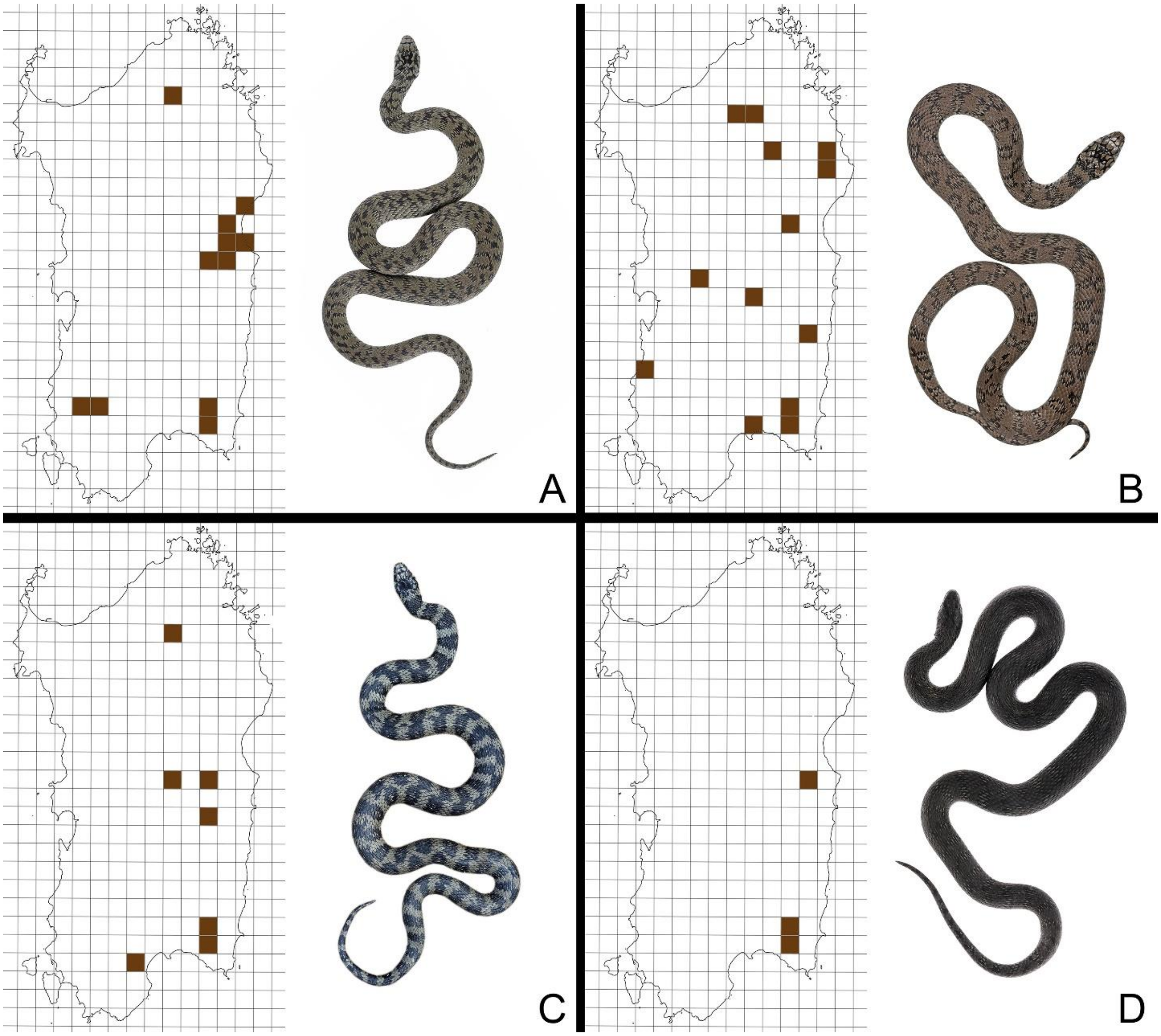

Figure S8. Distribution of the four different *Natrix helvetica cetti* dorsal patterns in the 10x10 km UTM map of Sardinia: category I (A), category II (B), category III (C) and category IV (D).
